# Multi-Omics Analysis Based on Genomic Instability for Prognostic Prediction in Lower-Grade Glioma

**DOI:** 10.3389/fgene.2021.758596

**Published:** 2022-01-05

**Authors:** Yudong Cao, Hecheng Zhu, Weidong Liu, Lei Wang, Wen Yin, Jun Tan, Quanwei Zhou, Zhaoqi Xin, Hailong Huang, Dongcheng Xie, Ming Zhao, Xingjun Jiang, Jiahui Peng, Caiping Ren

**Affiliations:** ^1^ Department of Neurosurgery, National Clinical Research Center for Geriatric Disorders, Xiangya Hospital, Central South University, Changsha, China; ^2^ Changsha Kexin Cancer Hospital, Changsha, China; ^3^ Key Laboratory for Carcinogenesis of Chinese Ministry of Health, School of Basic Medical Science, Cancer Research Institute, Central South University, Changsha, China; ^4^ Department of Medical Ultrasonics, Seventh Affiliated Hospital of Sun Yat-Sen University, Shenzhen, China

**Keywords:** genome instability, lower-grade glioma, multi-omics analysis, long noncoding RNA, signature

## Abstract

**Background:** Lower-grade gliomas (LGGs) are a heterogeneous set of gliomas. One of the primary sources of glioma heterogeneity is genomic instability, a novel characteristic of cancer. It has been reported that long noncoding RNAs (lncRNAs) play an essential role in regulating genomic stability. However, the potential relationship between genomic instability and lncRNA in LGGs and its prognostic value is unclear.

**Methods:** In this study, the LGG samples from The Cancer Genome Atlas (TCGA) were divided into two clusters by integrating the lncRNA expression profile and somatic mutation data using hierarchical clustering. Then, with the differentially expressed lncRNAs between these two clusters, we identified genomic instability-related lncRNAs (GInLncRNAs) in the LGG samples and analyzed their potential function and pathway by co-expression network. Cox and least absolute shrinkage and selection operator (LASSO) regression analyses were conducted to establish a GInLncRNA prognostic signature (GInLncSig), which was assessed by internal and external verification, correlation analysis with somatic mutation, independent prognostic analysis, clinical stratification analysis, and model comparisons. We also established a nomogram to predict the prognosis more accurately. Finally, we performed multi-omics-based analyses to explore the relationship between risk scores and multi-omics data, including immune characteristics, *N* ^6^-methyladenosine (m^6^A), stemness index, drug sensitivity, and gene set enrichment analysis (GSEA).

**Results:** We identified 52 GInLncRNAs and screened five from them to construct the GInLncSig model (CRNDE, AC025171.5, AL390755.1, AL049749.1, and TGFB2-AS1), which could independently and accurately predict the outcome of patients with LGG. The GInLncSig model was significantly associated with somatic mutation and outperformed other published signatures. GSEA revealed that metabolic pathways, immune pathways, and cancer pathways were enriched in the high-risk group. Multi-omics-based analyses revealed that T-cell functions, m^6^A statuses, and stemness characteristics were significantly disparate between two risk subgroups, and immune checkpoints such as PD-L1, PDCD1LG2, and HAVCR2 were significantly highly expressed in the high-risk group. The expression of GInLncSig prognostic genes dramatically correlated with the sensitivity of tumor cells to chemotherapy drugs.

**Conclusion:** A novel signature composed of five GInLncRNAs can be utilized to predict prognosis and impact the immune status, m^6^A status, and stemness characteristics in LGG. Furthermore, these lncRNAs may be potential and alternative therapeutic targets.

## Introduction

Gliomas originating primarily from progenitor glial cells are the most widely investigated malignant neoplasm in the central nervous system (CNS) and are accompanied by ascendent morbidity and mortality rates (Nava-Salazar et al., 2018; Li et al., 2019a). Currently, the WHO assorted gliomas into different grades and identified grade II and III gliomas as lower-grade glioma (LGG) and grade IV as high-grade glioma, i.e., glioblastoma (GBM) (Wu et al., 2020). In contrast to GBM, LGG behaves in a more sluggish course (Binder et al., 2019). Despite the development in the integrated treatment of LGG, including neurosurgical resection, chemo- and radiotherapy, targeted therapy, and immunotherapy, neoplasm relapse and malignant transformation to GBM are unavoidable due to their highly invasive property (Brat et al., 2015; Tan et al., 2020). Furthermore, since the survival time of the LGG patients ranges extensively from 1 to 15 years, the short-term or long-term survival of LGG patients cannot be accurately estimated (Nikas, 2014; Brat et al., 2015). Therefore, how to enhance the therapeutic effect and accuracy of predicting prognosis is paramount for LGG patients.

Genomic instability is defined as a high-frequency alteration in DNA (Murcia et al., 2019). Human genomic stability is maintained by various mechanisms, such as DNA damage responses, mitotic segregation mechanisms, and cell cycle checkpoints, and any defects in the operation of these mechanisms may lead to increased genomic vulnerability (Vincent et al., 2014). Genomic instability, manifesting in three primary forms (nucleotide, chromosomal, or microsatellite instability), contributes to the tumorigenesis and heterogeneity of diversified kinds of tumors and has emerged as one of the most crucial predictive elements for survival in many cancers, such as lung cancer and colorectal cancer (Jin and Burkard, 2018; Zhang et al., 2020a). Therefore, it is essential to identify the potential molecular features of genomic instability in various tumors and explore their associated clinical significance.

Noncoding RNAs, including long noncoding RNAs (lncRNAs; more than 200 nucleotides) and microRNAs (miRNAs; 19–25 nucleotides), account for more than 90% of the transcriptome and do not have protein-coding potential (Huarte, 2015). Aberrant expression of lncRNAs is usually associated with cancer development or progression (Li et al., 2017). Emerging evidence suggested that lncRNAs play essential regulatory roles in cell proliferation, differentiation, invasion, migration, and apoptosis (Gupta et al., 2010; Lee et al., 2016; Leucci et al., 2016). In addition, recent studies have shown that lncRNAs can regulate the expression of some crucial tumor suppressors or oncogenes through lncRNA–mRNA or lncRNA–miRNA interactions to affect tumorigenesis and progression (Gomes et al., 2013). Moreover, growing evidence disclosed the pivotal role of lncRNAs in regulating genomic stability. For instance, Hu et al. (2018) found a p53-responsive lncRNA GUARDIN, which was necessary to maintain genomic stability by promoting DNA damage repair. Lee et al. (2016) demonstrated that a highly conserved and abundant lncRNA (LINC00657) was activated after DNA damage and involved in maintaining genome stability by isolating some proteins that can hyperactively inhibit mitosis, DNA repair, and DNA replication. LncRNA MALAT1 promoted DNA repair by acting as a scaffold that directly interplayed with DNA repair proteins such as PARP1 and LIG3 (Lin et al., 2007). Although certain lncRNAs have been associated with genomic stability, sequence-based studies that systematically assess lncRNAs related to genomic instability and their clinical implications in LGG patients remain scarce.

In the present study, we applied single-nucleotide variant (SNV) and transcriptome profiling data to develop a lncRNA signature associated with genomic instability and investigated its prognostic value in patients with LGG. Next, we confirmed the effectiveness of the prediction model with internal and external datasets. Functional enrichment analysis was also performed to investigate its underlying mechanisms. Moreover, we also explored the relationships between risk score and tumor chemoresistance, *N* ^6^-methyladenosine (m^6^A) mRNA status, stemness index, and immune characteristics to provide a fresh perspective on predicting prognosis and treatment strategies for patients with LGG.

## Materials and Methods

### Study Flowchart

**FIGURE 1:**
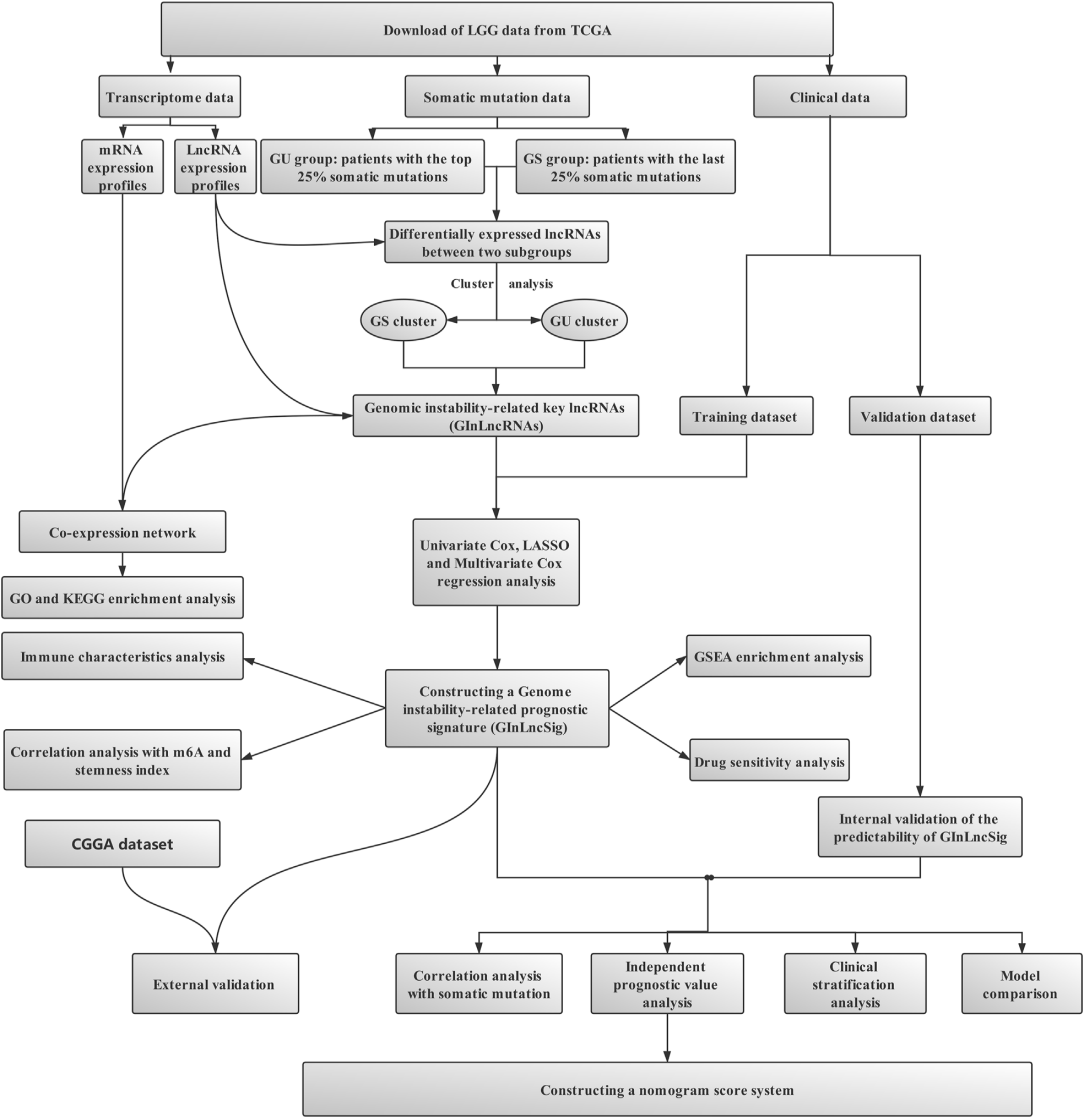
Flowchart for data collection and processing.

The main steps of this study are shown in Figure 1. After the data collection, the accumulated data of somatic mutation of each patient were counted. Then the top 25% and last 25% somatic mutation patients were extracted and divided respectively into genome unstable-like (GU) and genome stable-like (GS) groups. Differential analysis of lncRNA expression profiles between these two subgroups was performed. Then, the unsupervised hierarchical clustering analysis of all the samples based on the differentially expressed lncRNAs between the GS and GU groups was conducted to identify the GS and GU clusters. The differentially expressed lncRNAs between the GS and GU clusters, namely, genomic instability-related InRNAs (GInLncRNAs), were identified. The potential functions and pathways of the GInLncRNAs were analyzed using the gene co-expression, Gene Ontology (GO), and Kyoto Encyclopedia of Genes and Genomes (KEGG) analysis. Subsequently, the entire The Cancer Genome Atlas (TCGA) samples were randomly divided into training and validation datasets in a 1:1 ratio. The prognostic lncRNA risk signature was constructed in combination with the GInLncRNAs and the training dataset by univariate Cox, least absolute shrinkage and selection operator (LASSO), and multivariate Cox regression analyses. The evaluation of this model was performed by correlation analysis of somatic mutations, independent prognostic value analysis, clinical stratification analysis, model comparison, and internal and external dataset validation. Additionally, joint analyses of the risk signature and other multi-omics data, including tumor chemoresistance, m^6^A methylation status, stemness index, immune characteristics, and gene set enrichment analysis (GSEA), were also investigated. In the end, a nomogram was established to predict the patient’s prognosis more accurately.

### Data Source and Preparation

The transcriptome data (fragments per kilobase million (FPKM)), somatic mutation data (VarScan2), and corresponding clinical features of LGG were downloaded from TCGA database (https://portal.gdc.cancer.gov/). Next, we took the intersection of the above three parts of data to get the common samples according to the sample ID and removed the patients with missing survival information or too short follow-up (less than 30 days) from our study to exclude the influence of noncancer cause of death. Finally, 477 patients with matching somatic mutation data, lncRNA and mRNA expression profiles, survival information, and clinical features were obtained for further analysis. Subsequently, the cohort of 477 patients with LGG in TCGA was randomly classified into training and validation datasets at a 1:1 ratio with the “caret” package in the R language. The training dataset containing 240 patients was used to establish a predictive risk model, namely, the genomic instability-related InRNAs signature (GInLncSig). The validation dataset containing 237 patients as internal validation was used to verify the efficiency of this predictive model. The clinical features of these two datasets are exhibited in Table 1 (*p* > 0.05, chi-square test).

**TABLE 1:** Clinicopathological characteristics of the patients with lower-grade glioma (LGG) in three datasets.

Covariates	Type	Training dataset	Validation dataset	Total	*p*-Value
Age	≤41	120 (50%)	126 (53.16%)	246 (51.57%)	0.5486
Age	>41	120 (50%)	111 (46.84%)	231 (48.43%)	
Gender	Female	102 (42.5%)	114 (48.1%)	216 (45.28%)	0.2556
Gender	Male	138 (57.5%)	123 (51.9%)	261 (54.72%)	
Grade	G2	121 (50.42%)	110 (46.41%)	231 (48.43%)	0.4076
Grade	G3	118 (49.17%)	127 (53.59%)	245 (51.36%)	
Grade	Unknown	1 (0.42%)	0 (0%)	1 (0.21%)	

Note. Chi-squared test; *p* < 0.05 suggests significant difference.

In addition, we downloaded the RNA-seq and corresponding clinical data of another independent glioma dataset (DataSet ID: mRNAseq_693) with 693 samples from the Chinese Glioma Genome Atlas (CGGA) for external validation. By filtering out the patients with no survival information and a follow-up of fewer than 30 days, there were remaining 656 patients used for our research.

### Hierarchical Clustering and Screen of Genomic Instability-Related LncRNAs

We used the “maftools” R package to analyze and visualize somatic mutation profiles (Mayakonda et al., 2018). For the transcript data, we draw on the Bao and Geng et al. (2021) mutator hypothesis-derived computational framework (Bao et al., 2020). Briefly, we calculated the cumulative counts of somatic mutation for each patient and then ranked each patient in the order of the numbers of somatic mutations from high to low. The top 25% of somatic mutation patients were assigned as the GU group; the last 25% were designated as the GS group. We first identified the differentially expressed lncRNAs between the GS and GU groups with the Wilcoxon rank-sum test in the “limma” package of the R language. |Log_2_ (Fold change)| > 1.585 and false discovery rate (FDR) adjusted *p* < 0.05 were considered as filtering criteria. The volcano of the above differential expression lncRNAs was plotted by the “limma” package in the R language. After Z-score normalization of the expression data of the above differentially expressed lncRNAs, we performed hierarchical clustering (“hcluster” function in R) to stratify all the 477 patients into two clusters within Euclidean distance by using “limma,” “sparcl,” and “pheatmap” package of R language. According to the median of somatic mutations of the above two clusters, the cluster with a higher median of somatic mutations was assigned as the GU cluster; on the contrary, another cluster with a lower median of somatic mutation was defined as the GS cluster. Subsequently, the genomic instability-related lncRNAs (GInLnRNAs) were identified by differential expression analysis between two genomic instability subclusters as previously described.

### Co-Expression Network and Functional Enrichment Analysis

The lncRNA–mRNA co-expression network analysis was conducted based on Pearson’s correlation analysis between expression data of the lncRNA and mRNA using the “limma” package in R language to predict the unknown function of mRNA co-expressed with GInLncRNAs. We selected the top 10 Pearson’s correlation coefficients of mRNA as co-expressed GInLncRNA-related partners and visualized the co-expression network using the “igraph” package in the R language. GO and KEGG analyses were performed based on the above-selected mRNA partners to explore the potential function and pathway of GInLncRNAs by applying the “org.Hs.eg.db,” “clusterProfiler,” “ggplot2,” and “enrichplot” packages of R language (Chen et al., 2017).

### Development and Evaluation of GInLncRNA-Based Prognostic Signature

Univariate Cox regression analysis of GInLncRNAs was performed in the training dataset (*n* = 240) to screen prognosis-related GInLncRNAs using the “survival” package in R language with *p*-value <0.05. Then, the “glmnet” and “survminer” packages in R language were applied for the LASSO regression analysis with 10-fold cross-validations based on the above prognostic GInLncRNAs to avoid multicollinearity. Subsequently, multivariate survival analysis was enforced using the Cox multivariate proportional hazards regression model to obtain an optimal predictive signature of GInLncRNAs with the following formula: GInLncSig (Risk core) = $\sum_{i=1}^{n}coefficient\left(GInLncRNA_{i}\right)\times \exp ression\left(GInLncRNA_{i}\right)$ . The GInLncRNA_i_ means the ith selected GInLncRNA.

We used the median value of the risk score in the training dataset as a risk cutoff to divide the LGG patients into high-risk (>median value) and low-risk (≤median value) groups. The Kaplan–Meier (KM) survival curve was drawn using the R package “survminer” (*p* < 0.05 considered significance). The receiver operating characteristic (ROC) curves and the area under the curve (AUC) values at 1, 3, and 5 years were calculated using the R package “timeROC” to evaluate the predictive performance of this risk model of GInLncRNAs. What is more, the validation dataset and the entire TCGA cohort of LGG were used to assess the performance of the GInLncSig.

### Correlation Analysis of Somatic Mutation and Risk Score

We implemented the Wilcoxon signed-rank test in the training, validation, and entire TCGA datasets to determine the relationship between somatic mutation and risk score by the “limma” package in the R language.

### Independent Prognostic Analysis and Construction of a Nomogram Model

The relationship between GInLncSig and other clinical features was estimated by univariate and multivariate independent prognostic analyses to validate the independence of GInLncSig from other critical clinical features in the training, validation, and entire TCGA datasets using the “survival” package in R language. Then, based on the result of multivariate Cox regression analysis, a nomogram was constructed in the training dataset to predict the prognosis of patients with LGG at 1-, 3-, and 5-year overall survival (OS) using the “survival” and “regplot” packages of R language.

### Clinical Stratification Analysis and Model Comparison

We performed a clinical stratification analysis to evaluate the stability of prediction of GInLncSig. Briefly, in the validation dataset, we classified the patients into subgroups according to clinical parameters, such as age with 41 years as the demarcation point, gender (male and female), and tumor grade (G2 and G3). Each subgroup was then divided into high- and low-risk groups according to the median GInLncSig score. Survival analysis was performed between high- and low-risk groups in each subgroup using KM and log-rank test by “survival” and “survminer” packages in R language. In addition, we searched the LncRNA prognostic model from previous studies. Multivariable Cox regression analyses were used to train the existing signatures according to their gene name in the same samples of the entire TCGA dataset. We compared the prediction accuracy at 3-year OS by plotting the corresponding ROC curve and calculating the AUC values.

### External Validation From the Chinese Glioma Genome Atlas Database

The GInLncSig was further evaluated by another specialized glioma database of CGGA. We obtained 656 samples with the matched expression of lncRNAs and clinical data from the above database (DataSet ID: mRNAseq_693). Then this cohort was divided into subgroups according to the clinical features, including age, gender, and tumor grade, as mentioned earlier. We extracted CRNDE of the GInLncRNAs from the GInLncSig model and compared its expression among different subgroups using the “limma” and “ggpubr” packages in R language. Besides, the entire CGGA cohort was divided into two subgroups according to the expression median of the selected LncRNA from the GInLncSig model, and survival analysis was conducted in these two subgroups using “survival” and “survminer” packages in R language.

### Associations Between GInLncSig and Immune Characteristics

Correlation analysis was performed to evaluate the GInLncSig’s ability to predict immune characteristics such as immune cell infiltration, immune function, and immune checkpoint expression. The LGG cohort of TCGA dataset was classified into high- and low-risk groups according to the median value of GInLncSig before immune characteristics analysis.

Immune cell abundance was estimated between high- and low-risk groups based on the GInLncSig using the R package “immunedeconv” (Sturm et al., 2019), which integrates seven state-of-the-art algorithms TIMER (Li et al., 2016), CIBERSORT (Newman et al., 2015), CIBERSORT-ABS (Li et al., 2020), quanTIseq (Finotello et al., 2019), MCP-counter (Becht et al., 2016), xCell (Aran et al., 2017), and EPIC (Racle et al., 2017). The significant differences in immune cell infiltration based on the above algorithms were shown using a heatmap at *p*-value <0.05. Single-sample GSEA (ssGSEA) was implemented to assess immune function using the “GSVA” package in R language with method specification as “ssgsea” (Hänzelmann et al., 2013). In addition, we compared the expression level of the eight immune checkpoints, considered as potential or existing targets for tumor immunotherapy, between the high- and low-risk groups.

### Gene Set Enrichment Analysis in GInLncSig

GSEA was implemented using GSEA software (http://www.broadinstitute.org/gsea) between high- and low-risk groups based on TCGA dataset with the KEGG gene sets. The enrichment pathways with FDR < 0.01 were chosen for the drawing of the enrichment diagram.

### Relationship Between GInLncSig and m^6^A as Well as Stemness Index

To investigate the correlation between m^6^A and the risk of GInLncSig, we acquired the list of the m^6^A-related genes from Li’s study about the molecular characterization and clinical significance of m^6^A modulators across 33 cancer types (Li et al., 2019b). The stemness index for TCGA was acquired from previous pan-cancer research (Malta et al., 2018). The relationship analysis of risk score and cancer stemness index was performed using Spearman’s correlation test.

### Drug Sensitivity Analysis

The CellMiner database comprising genomics and pharmacology information of 60 different tumor cell lines was accessed by the address (https://discover.nci.nih.gov/cellminer). In addition, Pearson’s test was conducted for correlational analysis between the expression of the LncRNAs from the GInLncSig and drug sensitivity. The correlational study was performed based on the effects of 792 medicines already approved by the Food and Drug Administration (FDA) or in clinical trials (Supplementary Table S1). The top 9 correlation coefficients were chosen for the plotting display.

### Statistical Analysis

The Mann–Whitney U test, a nonparametric test, was applied to compare two independent and continuous variables. All multiple comparisons were Bonferroni corrected. A chi-squared test was used for categorical variables. Two-tailed *p* < 0.05 was set as the threshold for statistical significance. All analyses and visualization were conducted using the R language (Version 4.0.2) with the corresponding functional package.

## Results

### Analysis of Mutation Profiles in Lower-Grade Glioma

**FIGURE 2:**
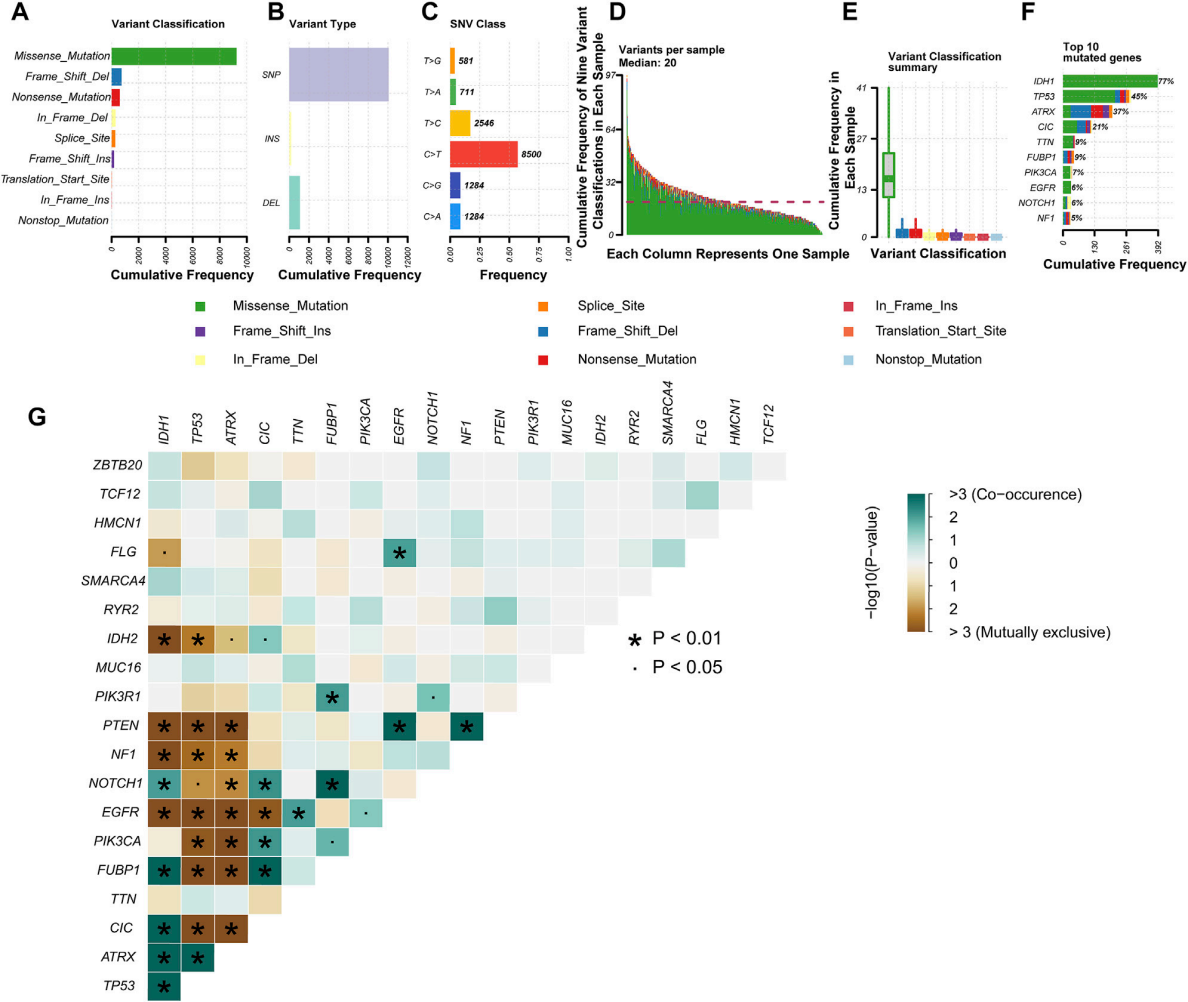
Profiles of mutation in patients with lower-grade glioma (LGG). Cohort summary plots show the distribution of variants based on variant classification **(A)**, type **(B)**, and single-nucleotide variant (SNV) class **(C)** according to LGG samples from The Cancer Genome Atlas (TCGA). **(A)** Frequency distribution histogram of the nine variant classifications. **(B)** Frequency distribution histogram of the three variant types (single-nucleotide polymorphism (SNP), INS, and DEL). **(C)** Frequency distribution histogram of the six base variant types. **(D)** Stacked histogram of the nine variant classifications in each LGG sample. **(E)** Box plot shows the frequency distribution of the nine variant classifications in each LGG sample. Various colors with specific annotations at the bottom part mean distinctive types of mutations (i.e., the nine variant classifications). **(F)** The stacked bar graph displays the top 10 mutated genes in LGG samples from TCGA. **(G)** Interaction between the top 20 mutated genes in LGG samples from TCGA.

The somatic mutation profiles of 477 LGG patients in the MAF format were acquired from TCGA database, and the data processed with VarScan2 software were selected for further analysis. We used the R package “maftools” to analyze and visualize the somatic mutation data. In the gross, the diversified mutations were grouped into different categories, where missense mutation dominates the mutation types (Figure 2A), single-nucleotide polymorphism (SNP) occupied a higher proportion than insertion or deletion (Figure 2B), and C > T happened more frequently than other SNVs in LGG (Figure 2C). In addition, we reckoned the numbers of variants per sample and exhibited mutation kinds by box plots with variant colors for LGG (Figures 2D,E). The top 10 mutated genes in LGG were presented in a horizontal histogram, including IDH1 (77%), TP53 (45%), ATRX (37%), CIC (21%), TTN (9%), FUBP1 (9%), PIK3CA (7%), EGFR (6%), NOTCH1 (6%) and NF1 (5%) (Figure 2F). Figure 2G exhibits the co-occurrence and mutually exclusive relation between mutated genes, and IDH1 mutations are often accompanied by a mutation in TP53 and ATRX. The overall mutation information for each sample in LGG was presented in a waterfall plot, in which different colors meant various mutated classifications (Supplementary Figure S1).

### Identification of Genomic Instability-Related Subtypes and LncRNAs in Lower-Grade Glioma Patients

**FIGURE 3:**
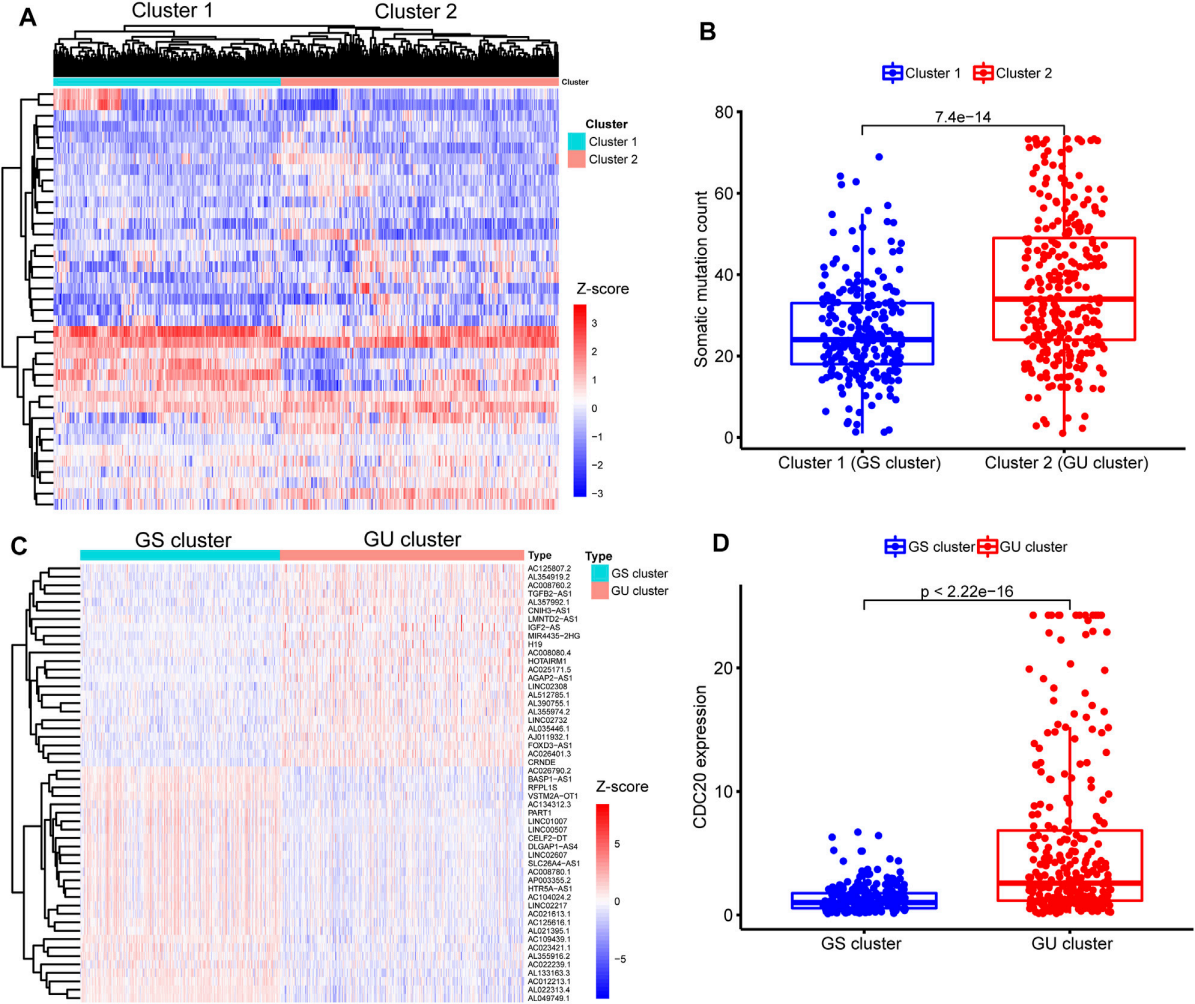
Identification of sub-clusters and long noncoding RNAs (lncRNAs) associated with genomic instability in patients with lower-grade glioma (LGG). **(A)** Heatmap of two genomic instability-derived sub-clusters (Cluster 1 and Cluster 2) based on unsupervised hierarchical clustering in entire The Cancer Genome Atlas (TCGA) samples. **(B)** Comparison for somatic mutations between Cluster 1 and Cluster 2. Fewer somatic mutations are presented in Cluster 1 than Cluster 2 (*p* < 0.001, Mann–Whitney U test). Hence, Cluster 1 was defined as the genomic stable (GS) cluster, while Cluster 2 was termed as the genomic unstable (GU) cluster. **(C)** Heatmap of expression of 52 genomic instability-related lncRNAs (GInLncRNAs) between the GS and GU clusters. The abscissa represents the LGG samples, classified into the GS cluster (blue) and GU cluster (red), and the ordinate is 52 GInLncRNAs. **(D)** Comparison of CDC20 expression levels between the GS cluster and GU cluster. Lower expression levels of CDC20 were seen in the GS cluster compared with the GU cluster (*p* < 0.001, Mann–Whitney U test).

To identify the genomic instability-derived subtypes, we first calculated the cumulative numbers of somatic mutations for each patient with LGG and ranged them in descending order. Then, we assigned the 133 samples with the top 25% somatic mutations as the GU group and 137 samples with the last 25% somatic mutations as the GS group. Thirty-nine lncRNAs were determined to be differentially expressed significantly between the above two groups according to the |Log_2_ (Fold change)| > 1.585 and FDR adjusted *p*-value <0.05 (Supplementary Figure S2). Subsequently, an unsupervised hierarchical clustering analysis assigned the entire TCGA cohort into two clusters (Cluster 1 and Cluster 2) based on the above 39 differentially expressed lncRNAs (Figure 3A). Cluster 2 with higher somatic mutations was called the GU cluster, and Cluster 1 with lower somatic mutations was termed the GS cluster (*p* < 0.001, Mann–Whitney U test; Figure 3B). KM survival analysis showed that LGG patients in the GU cluster had a significantly poorer prognosis than those in the GS cluster (*p* < 0.001, log-rank test; Supplementary Figure S3).

Next, we identified 52 differently expressed lncRNAs termed GInLncRNAs between the GS and GU clusters. There were 24 upregulated and 28 downregulated lncRNAs in the GU cluster compared with the GS cluster (Figure 3C and Supplementary Table S2).

Besides, we found that CDC20 gene, one of the recently identified markers of genomic instability in glioma, was significantly upregulated in the GU cluster (*p* < 0.001, Mann–Whitney U test; Figure 3D) (Zhang et al., 2019). These results indicated that our selected 52 lncRNAs could be regarded as matriculant GInLncRNAs.

### Co-Expression and Enrichment Analysis

**FIGURE 4:**
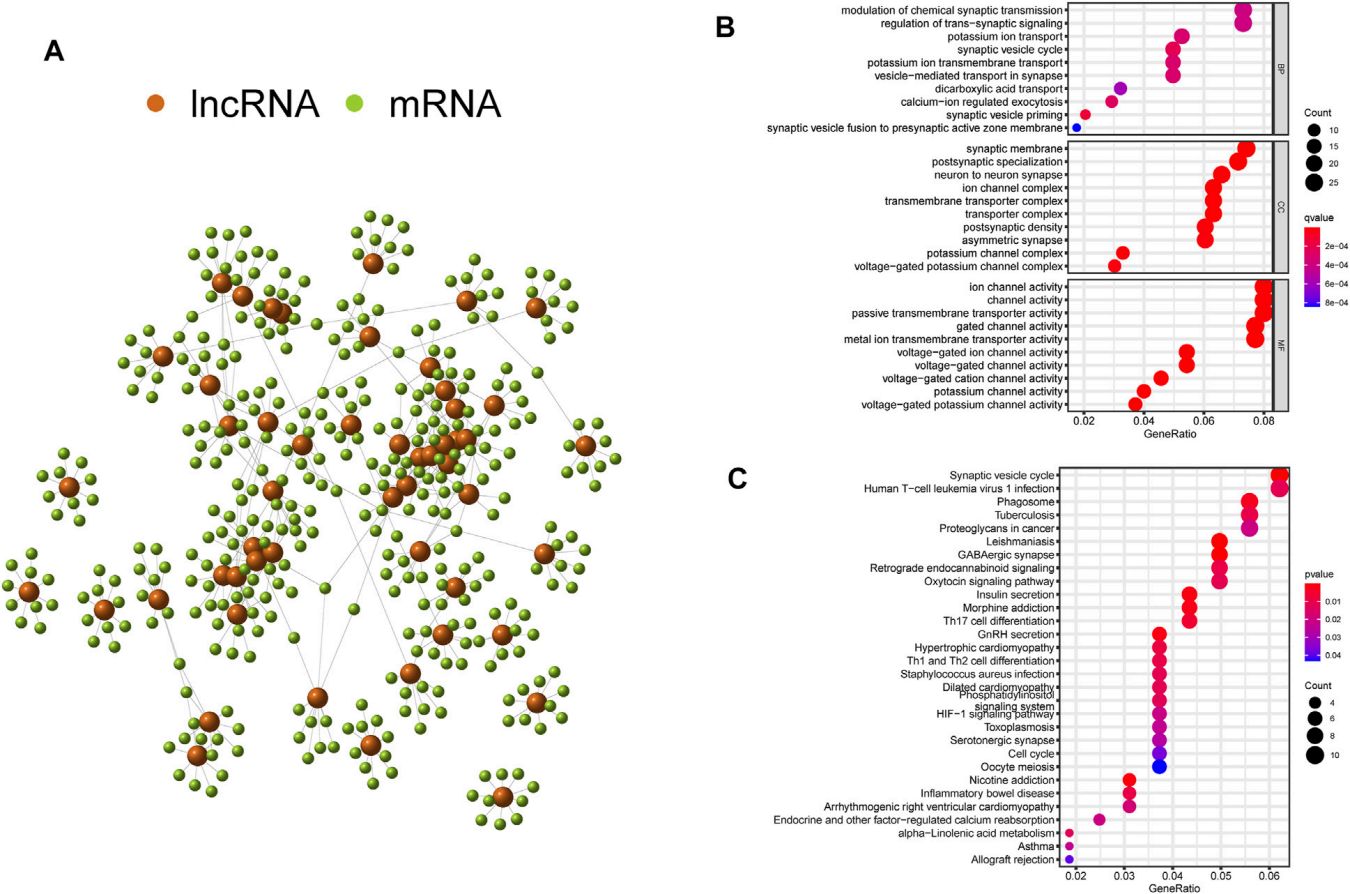
Co-expression network and enrichment analyses of genomic instability-related long noncoding RNAs (lncRNAs) (GInLncRNAs). **(A)** Interaction relationship of GInLncRNAs and their top 10 co-expressed mRNAs based on Pearson’s correlation coefficients. **(B)** Bubble plots of Gene Ontology (GO) and **(C)** Kyoto Encyclopedia of Genes and Genomes (KEGG) enrichment analyses of co-expressed mRNAs with GInLncRNAs.

To explore the potential functions and pathways of the GInLncRNAs, we performed a co-expression analysis between the GInLncRNAs and mRNAs and acquired the top 10 mRNAs most related to each GInLncRNA according to Pearson’s correlation coefficient. As shown in Figure 4A, the lncRNAs and mRNAs were represented by nodes of two colors, and the correlative lncRNA–mRNAs were connected. Then the GO and KEGG enrichment analyses of the mRNAs co-expressed with GInLncRNAs were conducted. The GO function enrichment analysis demonstrated that the GInLncRNA-related mRNAs markedly enriched in synaptic signaling transmission and regulation in the biological process (BP), synaptic membrane in the cellular component (CC), and ion channel activity in the molecular function (MF; adjusted *p*-values <0.05, Figure 4B and Supplementary Table S3). In terms of the KEGG pathway enrichment analysis, 35 significantly enriched pathways were obtained (Supplementary Table S4). These enriched pathways are markedly involved in neuro-synaptic signaling pathways, immune signaling pathways, cancer signaling pathways, and so on (Figure 4C). These results implied that the changes in GInLncRNA expression might influence the transmission of synaptic signals, immune status, and tumorigenesis by interfering with the counterpoise of the regulatory network of the mRNA co-expressed with the GInLncRNAs.

### Identification and Construction of Genomic Instability-Derived Prognostic Signature

**FIGURE 5:**
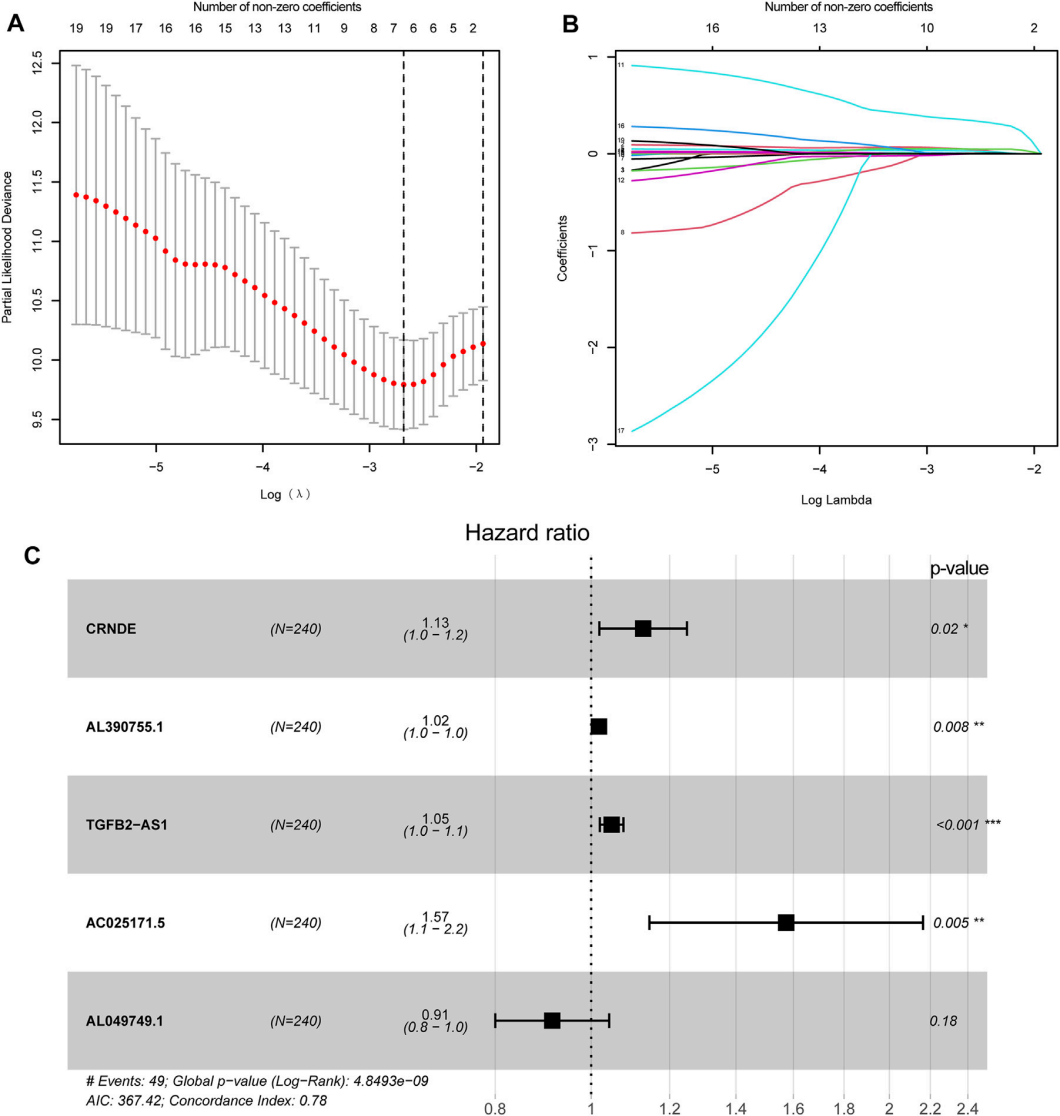
Construction of genomic instability-related lncRNA signature (GInLncSig). **(A)** Least absolute shrinkage and selection operator (LASSO) deviance profiles. Selecting λ value by 10-fold cross-validation. The λ value of −2.7 was chosen by 10-fold cross-validation with the minimum partial likelihood deviation, and seven variables were retained. **(B)** Processes of LASSO Cox model fitting. Seven variables were kept when the λ value was equal to −2.7. **(C)** The forest chart for the five lncRNA prognostic signature based on the stepwise multivariate Cox proportional hazards regression model. CRNDE, AL390755.1, TGFB2-AS1, and AC025171.5 were risk factors, while AL049749.1 was a protective factor.

**FIGURE 6:**
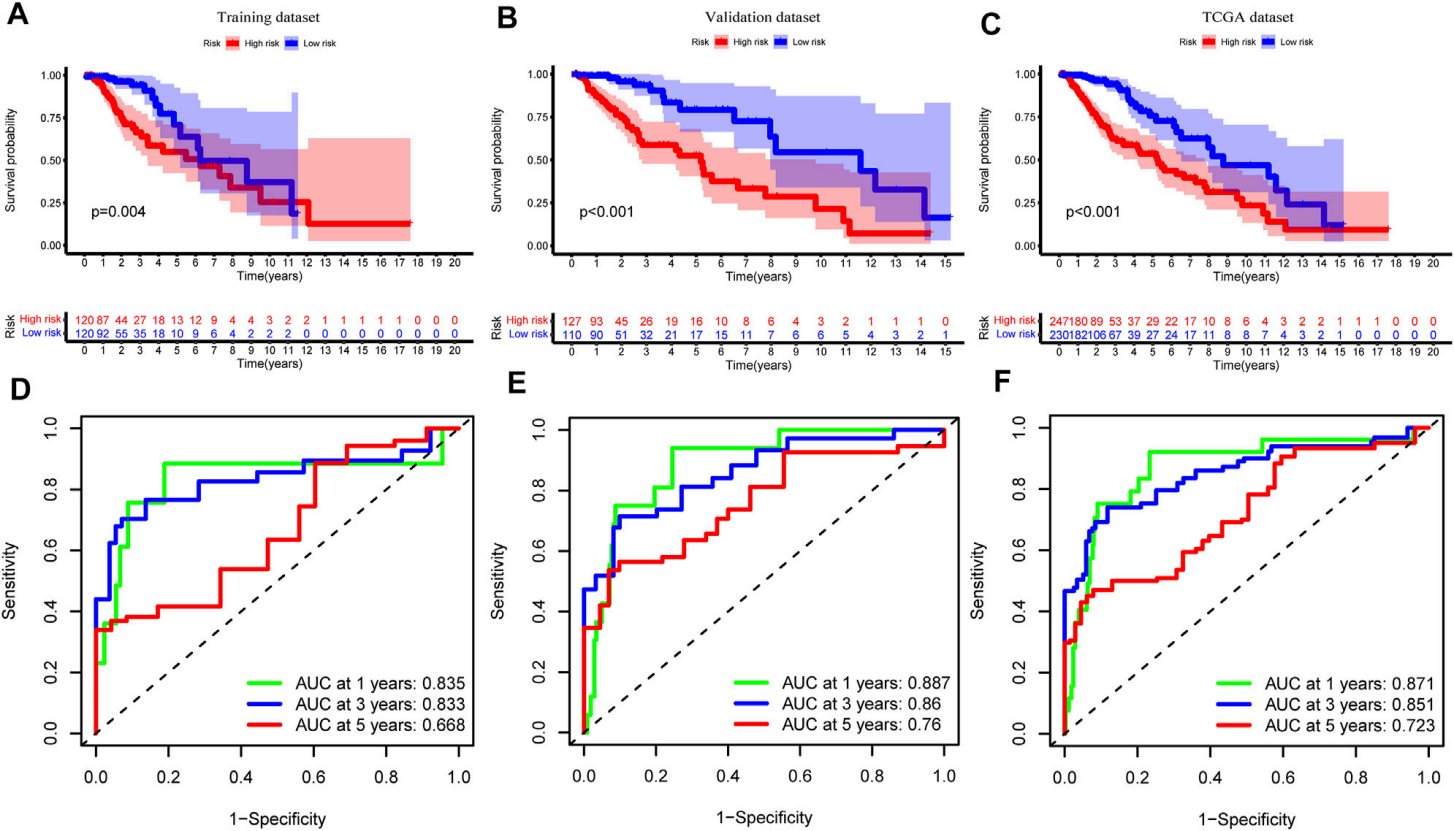
Assessment and verification of genomic instability-related long noncoding RNAs (lncRNA) signature (GInLncSig). Kaplan–Meier Survival analyses of GInLncSig for high- and low-risk groups in training **(A)**, validation **(B)**, and The Cancer Genome Atlas (TCGA) datasets **(C)**. Patients in the high-risk group display shorter overall survival (OS) than low-risk patients. Receiver operating characteristic (ROC) curve at 1, 3, and 5 years for survival prediction of GInLncSig in the training **(D)**, validation **(E)**, and TCGA datasets **(F)**.

To explore the association between the expression level of GInLncRNAs and clinical significance, we randomly assorted the 477 patients with LGG into two parts: the training dataset (*n* = 240) and the validation dataset (*n* = 237). No significant differences were discovered with the chi-square test in the distribution of basic clinical features among the training, validation, and entire TCGA datasets (*p* > 0.05, Table 1). To screen the GInLncRNAs related to prognosis, we first conducted a univariate Cox regression analysis in the training dataset and found that 26 of 52 GInLncRNAs were significantly associated with the OS of LGG (*p* < 0.05, Supplementary Table S5). Next, LASSO regression and multivariate Cox proportional hazards regression analysis in a stepwise manner were carried out among the screened 26 GInLncRNAs to create a prognostic model for survival prediction. Eventually, 5 of the 26 GInLncRNAs that reserved prognostic significance were identified in the genomic instability-related risk model (Figure 5). The genomic instability-derived lncRNA signature (GInLncSig) was built to evaluate the prognostic risk of LGG patients based on the expression levels of the above five independent prognostic GInLncRNAs and their coefficients of multivariate Cox analysis as the following formula: risk score (GInLncSig) = (0.121 × Exp_CRNDE_) + (0.018 × Exp_AL390755.1_) + (0.048 × Exp_TGFB2-AS1_) + (0.453 × Exp_AC025171.5_) + (−0.091 × Exp_AL049749.1_). In this GInLncSig, the coefficients of four LncRNAs (CRNDE, AL390755.1, TGFB2-AS1, and AC025171.5) were positive, indicating that they were risk factors and their high levels of expression predicted poor prognosis of LGG. In contrast, the lncRNA AL049749.1 with a negative coefficient served as a protective factor, and its high expression was an indicator of a better prognosis. The risk scores of 240 patients in the training dataset were calculated according to the GInLncSig. Then, using the median-risk score of 0.802 from the training dataset as the cutoff, these 240 patients in the training dataset were classified into the high-risk group (risk scores equal to or greater than the cutoff) and low-risk group (risk scores below the cutoff; Supplementary Table S6). Kaplan–Meier survival analysis showed that patients with LGG in the low-risk group had a significantly better OS than those in the high-risk group (*p* < 0.01, log-rank test; Figure 6A). Furthermore, the AUC values of the time-dependent ROC curves at 1, 3, and 5 years in the training dataset were 0.835, 0.833, and 0.668, respectively (Figure 6D).

To verify the predictive performance of the GInLncSig, we reckoned the risk scores of the validation dataset (*n* = 237) and the entire TCGA cohort (*n* = 477) and carried on the Kaplan–Meier survival analysis, and we plotted the corresponding ROC curves. The survival time of patients in the low-risk group was significantly longer than that in the high-risk group in the validation dataset and the entire TCGA cohort (*p* < 0.001, log-rank test; Figures 6B,C and Supplementary Tables S7, 8). The AUC values of ROC curves at 1, 3, and 5 years were respectively 0.887, 0.860, and 0.760 in the validation (Figure 6E), and 0.871, 0.851, and 0.723 in the entire TCGA cohort (Figure 6F). Altogether, these results suggested that the GInLncSig has an excellent predictive value for survival.

### Correlation Analysis Between the GInLncSig and Somatic Mutation Pattern

**FIGURE 7:**
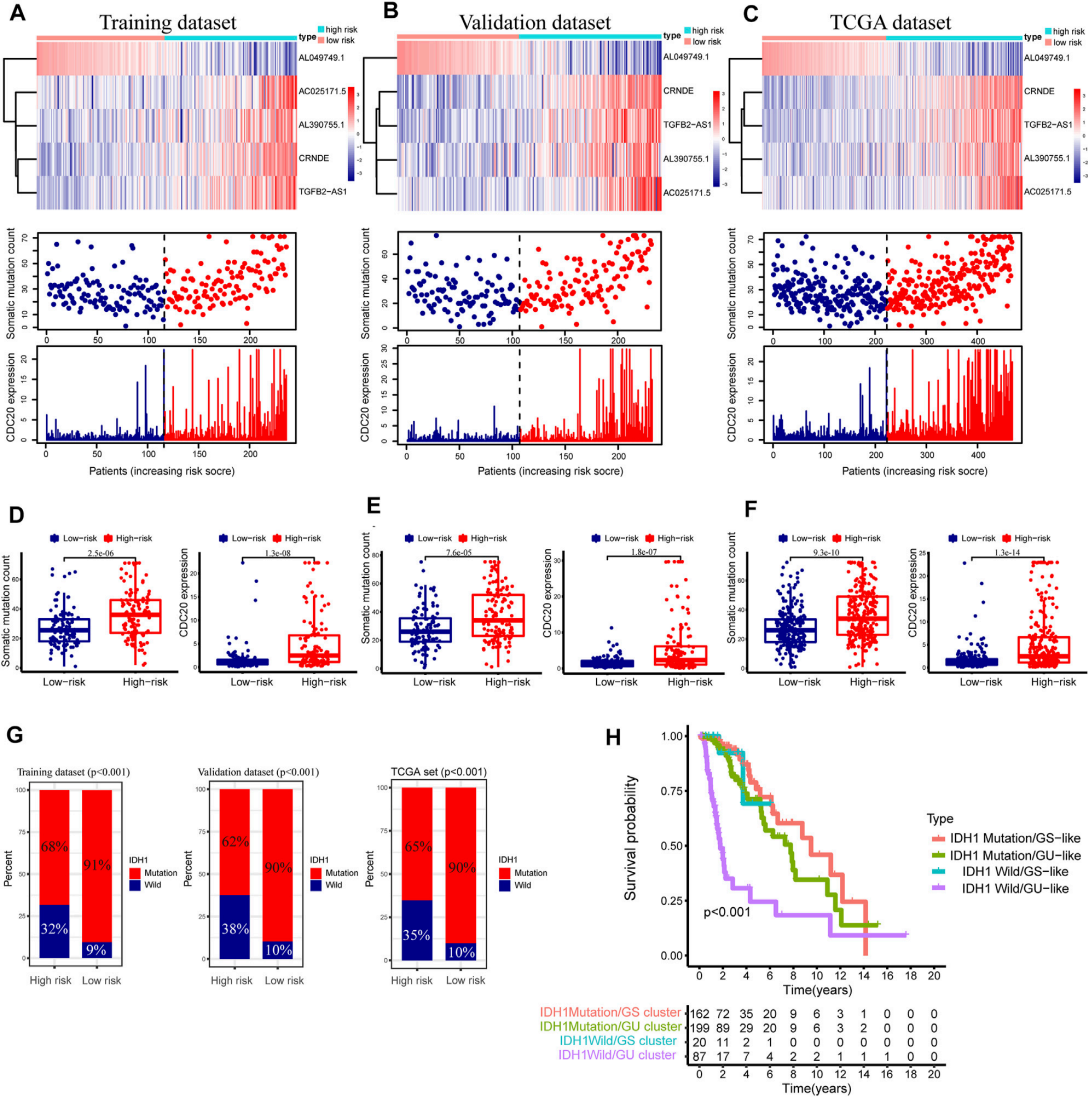
Relationship between the GInLncSig and somatic mutation and CDC20 expression level in three datasets. Heatmap of long noncoding RNAs (lncRNA) expression, the distribution of somatic mutation, and CDC20 expression with increasing risk score of patients in the training dataset **(A)**, validation dataset **(B)**, and The Cancer Genome Atlas (TCGA) dataset **(C)**. The Mann–Whitney U test was performed to compare the cumulative somatic mutations and CDC20 expression between the high- and low-risk groups for the training dataset **(D)**, validation dataset **(E)**, and TCGA dataset **(F)**. **(G)** The percentage of IDH1 mutation between the high- and low-risk groups in the training dataset, the validation dataset, and TCGA dataset (chi-squared test, *p* < 0.01). **(H)** Kaplan–Meier survival analyses were performed for patients grouped according to IDH1 mutation status and the genomic stable status. The overall survival outcomes of these four groups were significantly different (log-rank test, *p* < 0.001).

We drew a series of risk plots in which the samples were sorted in increasing order of risk scores for the training, validation, and entire TCGA datasets, including the heatmap of GInLncRNA expression, the distribution of somatic mutation of LGG patients, and the change of CDC20 expression pattern along with the increasing scores. As illustrated in Figure 7A, the expression levels of these risky lncRNAs (CRNDE, AL390755.1, TGFB2-AS1, and AC025171.5) in the training dataset were upregulated along with the increase of risk scores as expected. In contrast, the protective lncRNA AL049749.1 was downregulated along with the rise in risk scores. In addition, the numbers of somatic mutation and pattern of CDC20 expression presented an upward trend with increasing risk scores. In the training datasets, the high-risk group is significantly greater than the low-risk group in the number of somatic mutations (median of somatic mutation counts 36 vs. 25.5, *p* < 0.001, Mann–Whitney U test; Figure 7D). The patients in the high-risk group of the training dataset tended to have a greater expression level of CDC20 gene than the low-risk group (median of CDC20 expression level 2.484 vs. 1.052, *p* < 0.001, Mann–Whitney U test; Figure 7D). We further probed into whether the GInLncSig exhibited similar performance in the validation and entire TCGA datasets. As described in Figures 7B,C, the same procedures were repeated in the validation and entire TCGA datasets, and these similar results were observed. The high-risk group exhibited significantly higher somatic mutations than the low-risk group in both the validation (median of somatic mutation numbers 34.0 vs. 26.0, *p* < 0.001, Mann–Whitney U test; Figure 7E) and entire TCGA datasets (median of somatic mutation numbers 34.0 vs. 26.0, *p* < 0.001, Mann–Whitney U test; Figure 7F). Likewise, it could be observed that the high-risk group presented a higher expression level of CDC20 compared with the low-risk group in both the validation (median of CDC20 expression level 2.315 vs. 1.207, *p* < 0.001, Mann–Whitney U test; Figure 7E) and entire datasets (median of CDC20 expression level 2.451 vs. 1.079, *p* < 0.001, Mann–Whitney U test; Figure 7F).

As is known, the mutation state of IDH1 plays a vital role in the LGG (Wesseling and Capper, 2018), and our study showed a 77% mutation rate of IDH1 in the entire TCGA cohort of LGG (*n* = 477, Figure 2A). Consequently, the relation between the GInLncSig and the mutation status of IDH1 was further evaluated in the training, validation, and entire TCGA datasets. As illustrated in Figure 7G, the patients with IDH1 mutation remarkably dominated the proportion in the low-risk group, while the patients with IDH1 wild status were dominant in the high-risk group in all three datasets (*p* < 0.001, chi-squared test; Figure 7G).

Survival analysis was further conducted in combination with the mutation status of IDH1 and the hierarchical clusters, including IDH1 Mutation/GS cluster, IDH1 Mutation/GU cluster, IDH1 Wild/GS cluster, and IDH1 Wild/GU cluster. As shown in Figure 7H, the Kaplan–Meier survival curve illustrated significant survival divergences among the four groups (*p* < 0.001, log-rank test). The patients in IDH1 Wild/GU cluster had the poorest prognosis (Figure 7H). These results presented in Figures 7G,H implied the GInLncSig was related to the mutation status of IDH1. Collectively, the above results suggested the risk score of GInLncSig correlated to the somatic mutation patterns.

### The Clinical Stratification Validation of the GInLncSig

**FIGURE 8:**
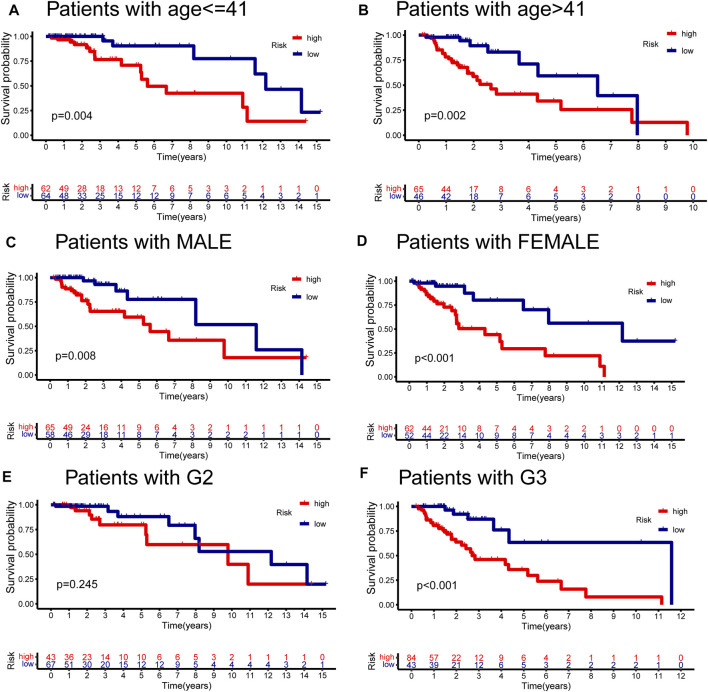
Stratified analyses of GInLncSig by age, gender, and tumor grade. Kaplan–Meier survival curves for high- and low-risk groups in the validation dataset with age ≤ 41 years **(A)**, age > 41 years **(B)**, gender in male **(C)**, gender in female **(D)**, tumor grade in II **(E)**, and tumor grade in III **(F)**. The high-risk patients displayed significantly worse overall survival (OS) than the low-risk patients across all clinical stratification subgroups except for the subset of patients with G2 (log-rank test, *p* < 0.05).

We conducted the stratified survival analysis of the validation dataset based on essential clinical information, including age, gender, and tumor grade. Figures 8A–F depicted the KM survival curve analyses, suggesting that patients in the low-risk group had significantly better survival prognoses than the high-risk group among all the clinical stratified subgroups (*p* < 0.01, log-rank test; Figures 8A–D,F) except the G2 subgroup (*p* = 0.245, log-rank test; Figure 8E).

### The GInLncSig Performs Better in Survival Prediction Than Other LncRNA-Related Signatures

**FIGURE 9:**
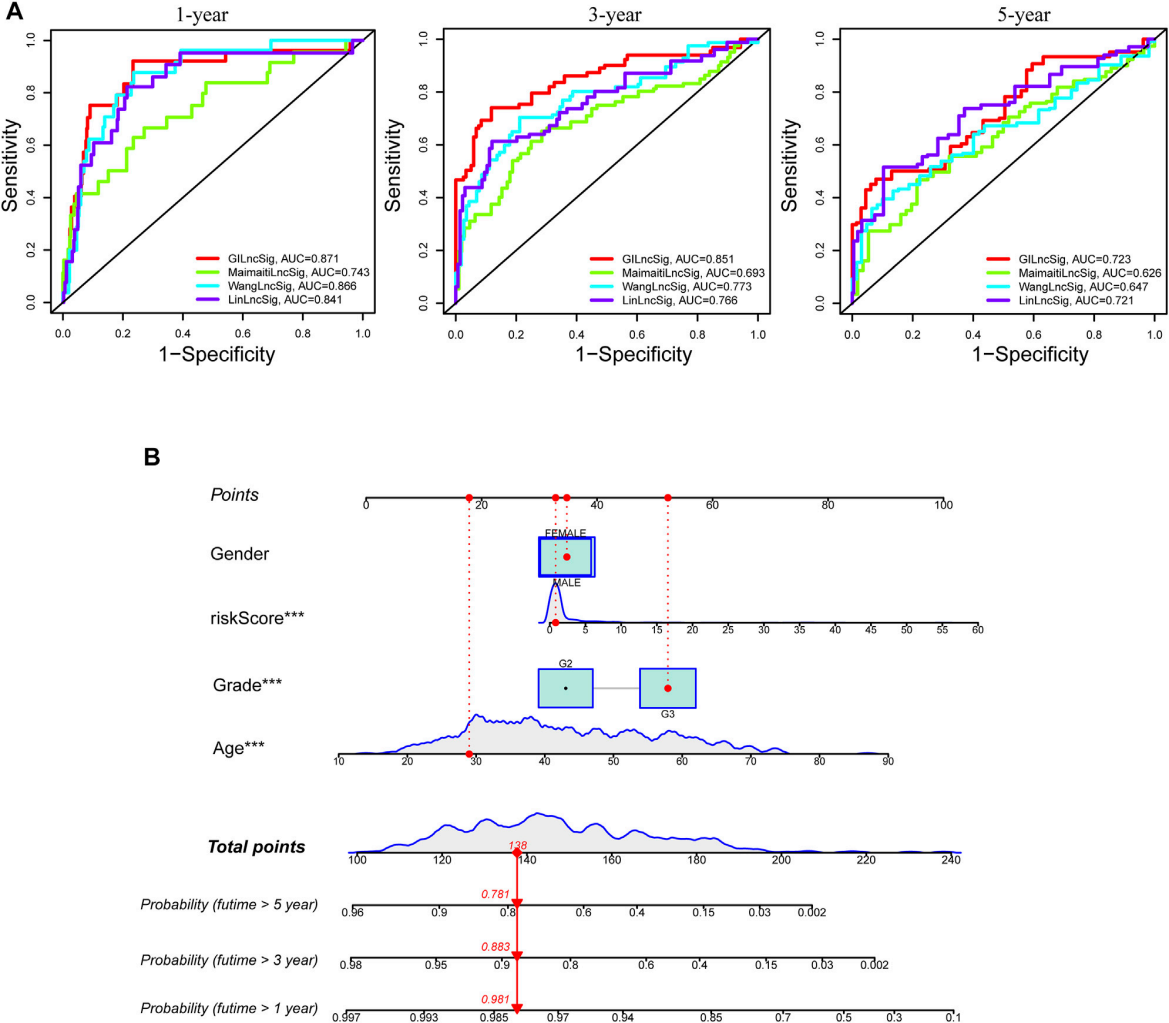
Comparison of survival prediction and construction of nomogram. **(A)** The receiver operating characteristic (ROC) curves for 1-, 3-, and 5-year survival prediction comparison between the GInLncSig and the other three existing signatures. **(B)** A nomogram was established integrating the GInLncSig, gender, age, and tumor grade, for predicting 1-, 3-, and 5-year survival outcomes of lower-grade glioma (LGG).

To compare the predictability of our GInLncSig model and the other three existing lncRNA-related signatures for LGG patients’ survival, we conducted ROC curve analyses using the same samples of the entire TCGA cohort. The other three lncRNA models were 8-lncRNA prognostic model documented by Maimaiti’s study (MaimaitiLncSig) (Maimaiti et al., 2021), 5-lncRNA predictive model derived from Wang’s study (WangLncSig) (Wang et al., 2020), and 6-lncRNA prognostic model reported by Lin’s study (LinLncSig) (Lin et al., 2020). As outlined in Figure 9A, our GInLncSig model (AUC = 0.871, 0.851, and 0.723 for 1, 3, and 5 years) outperformed the MaimaitiLncSig (AUC = 0.743, 0.693, and 0.626 for 1, 3, and 5 years), WangLncSig (AUC = 0.866, 0.773, and 0.647 for 1, 3, and 5 years), and LinLncSig (AUC = 0.841, 0.766, and 0.721 for 1, 3, and 5 years) in predicting 1-, 3- and 5-year OS of LGG patients. Moreover, there were five lncRNAs in our GInLncSig model, which were less than those in LinLncSig and MaimaitiLncSig (six and eight, respectively). Overall, the ROC curve results showed that for LGG patients, the GInLncSig could be more predictive of survival probability.

### Independent Prognostic Analysis of GInLncSig and Construction of a Nomogram

We carried out univariate and multivariate Cox regression analyses of age, gender, tumor grade, and GInLncSig in the training, validation, and entire TCGA datasets to identify the independent prognostic value of GInLncSig. After univariate risk factor analysis, the age (*p* < 0.05), tumor grade (*p* < 0.05), and risk score (*p* < 0.05) were correlated to survival and then retained in the multivariate analysis of risk factors. After multivariate Cox regression analysis, as shown in Table 2, age, tumor grade, and risk score had the independent prognostic value to the LGG patients across the three datasets.

**TABLE 2:** Independent prognostic analyses for risk score in three datasets.

Variables	Univariable Cox analysis			*p*-Value	Multivariable Cox analysis			*p*-Value
	HR	HR: 95% CI lower	HR: 95% CI higher		HR	HR: 95% CI lower	HR: 95% CI higher	
Training dataset (*n* = 240)								
Age	1.068	1.042	1.095	<0.001	1.062	1.035	1.091	<0.001
Gender	1.389	0.777	2.485	0.268				
Grade	2.482	1.364	4.514	0.003	1.894	1.013	3.544	0.046
Risk score	1.105	1.073	1.139	<0.001	1.085	1.052	1.119	<0.001
Validation dataset (*n* = 237)								
Age	1.062	1.041	1.085	<0.001	1.055	1.032	1.078	<0.001
Gender	0.794	0.473	1.334	0.383				
Grade	3.817	2.082	6.998	<0.001	2.805	1.490	5.282	0.001
Risk score	1.068	1.044	1.093	<0.001	1.046	1.020	1.073	<0.001
TCGA dataset (*n* = 477)								
Age	1.064	1.048	1.081	<0.001	1.057	1.040	1.074	<0.001
Gender	1.026	0.700	1.502	0.896				
Grade	3.056	2.015	4.634	<0.001	2.273	1.472	3.512	<0.001
Risk score	1.081	1.062	1.100	<0.001	1.059	1.039	1.079	<0.001

Note. HR, hazard ratio; TCGA, The Cancer Genome Atlas.

To improve the clinical practicability of the GInLncSig model, we constructed a predictive nomogram model encasing clinicopathological features and risk score in TCGA dataset. Figure 9B shows that the risk score was the dominant predictor.

### External Validation of One LncRNA Extracted From GInLncSig Model

To conduct a cross-platform validation of the GInLncSig, we used another independent dataset—mRNAseq_693 from the CGGA database. We found that only one (CRNDE) of five lncRNAs in the GInLncSig was covered by the mRNAseq_693 dataset because of the different depths of detection in various platforms. Therefore, we explored the association of CRNDE with clinicopathological characteristics and survival prognosis of LGG in the independent CGGA database (DataSet ID: mRNAseq_693).

**FIGURE 10:**
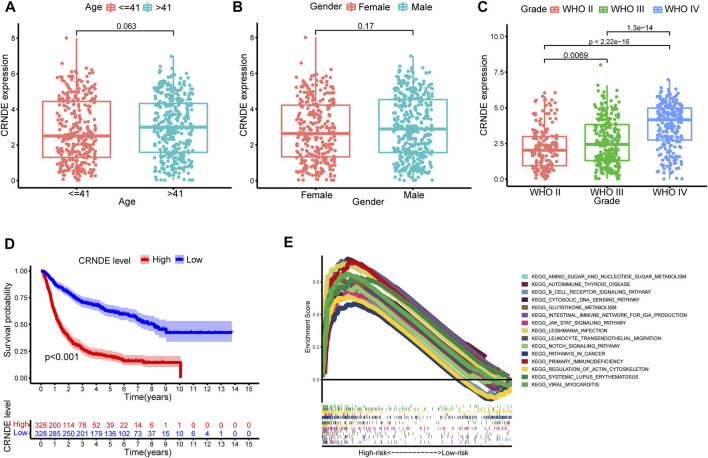
External validation of the predictive efficiency of CRNDE from GInLncSig and gene set enrichment analysis (GSEA). Relationship between CRNDE expression and age **(A)**, gender **(B)**, and tumor grade **(C)** in Chinese Glioma Genome Atlas (CGGA) dataset. Mann–Whitney U test. **(D)** Kaplan–Meier survival analysis between expression of CRNDE and overall survival (OS) in CGGA database (log-rank test, *p* < 0.001). **(E)** GSEA of pathways between high- and low-risk groups in The Cancer Genome Atlas (TCGA) dataset.

Figures 10A–C show that CRNDE expression was significantly related to tumor grade but not significantly associated with age (≤41 and >41 years old) and gender. Bonferroni-corrected pairwise comparisons indicated that the expression level of CRNDE increased substantially with the ascending sequence of tumor grade (median 2.011 vs. 2.341 vs. 4.135 in WHO II, III, and IV, respectively; Figure 10C). In addition, the KM survival analysis displayed a significantly worse prognosis for patients with high CRNDE expression levels consistent with CRNDE as a risk factor in the GInLncSig model (*p* < 0.001, log-rank test; Figure 10D).

### Associations Between GInLncSig and Immune Characteristics, and Gene Set Enrichment Analysis in GInLncSig

**FIGURE 11:**
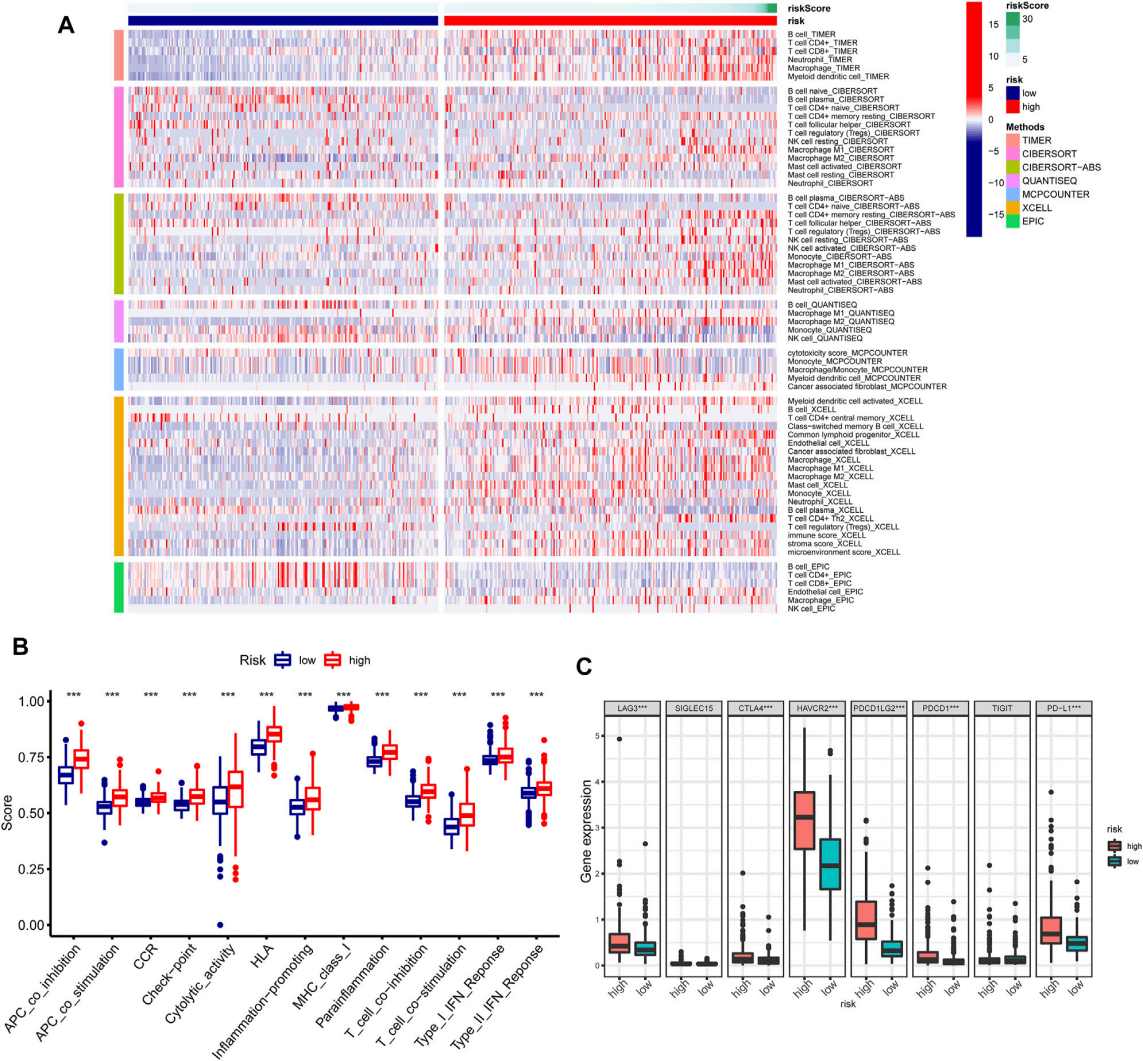
Immune characteristics analysis of GInLncSig in The Cancer Genome Atlas (TCGA) dataset. **(A)** Heatmap for immune cell infiltration based on TIMER, CIBERSORT, CIBERSORT-ABS, quanTIseq, MCP-counter, xCell, and EPIC algorithms between high- and low-risk groups. **(B)** Single-sample gene set enrichment analysis (GSEA) (ssGSEA) for the association between immune functions and GInLncSig. **(C)** The expression level of immune checkpoints between high- and low-risk groups.

The heatmap revealed the distribution of significantly different tumor-infiltrating immune cells estimated by multiple algorithms between the high- and low-risk groups in the entire TCGA cohort (Figure 11A). The relevance analysis of activity status of immune functions or cells based on ssGSEA score of TCGA dataset illustrated that the immune function scores of the high-risk group remarkably increased than those of the low-risk group in the antigen presentation function (APC co-inhibition and stimulation and MHC class I), T-cell functions (checkpoint, cytolytic activity, HLA, co-inhibition, and inhibition), inflammation regulation (inflammation-promoting and parainflammation), and so on (Figure 11B). Considering the immune checkpoint playing a vital role in immunotherapy, we further compared the expression of eight genes related to immune checkpoints between these two groups. The result suggested that immune checkpoint genes including LAG3, CTLA4, HAVCR2, PDCD1LG2, PDCD1, and PD-L1 were dramatically upregulated in the high-risk group except for SIGLEC15 and TIGIT (Figure 11C). The GSEAs presented the top 15 pathways with FDR values of less than 0.05 significantly enriched in the high-risk group, which were related to metabolic pathways, immune pathways, cancer pathways, and so on; however, the low-risk group had no significantly enriched items with FDR value of less than 0.05 (Figure 10E).

### Correlation of GInLncSig With m^6^A and Stemness Index

**FIGURE 12:**
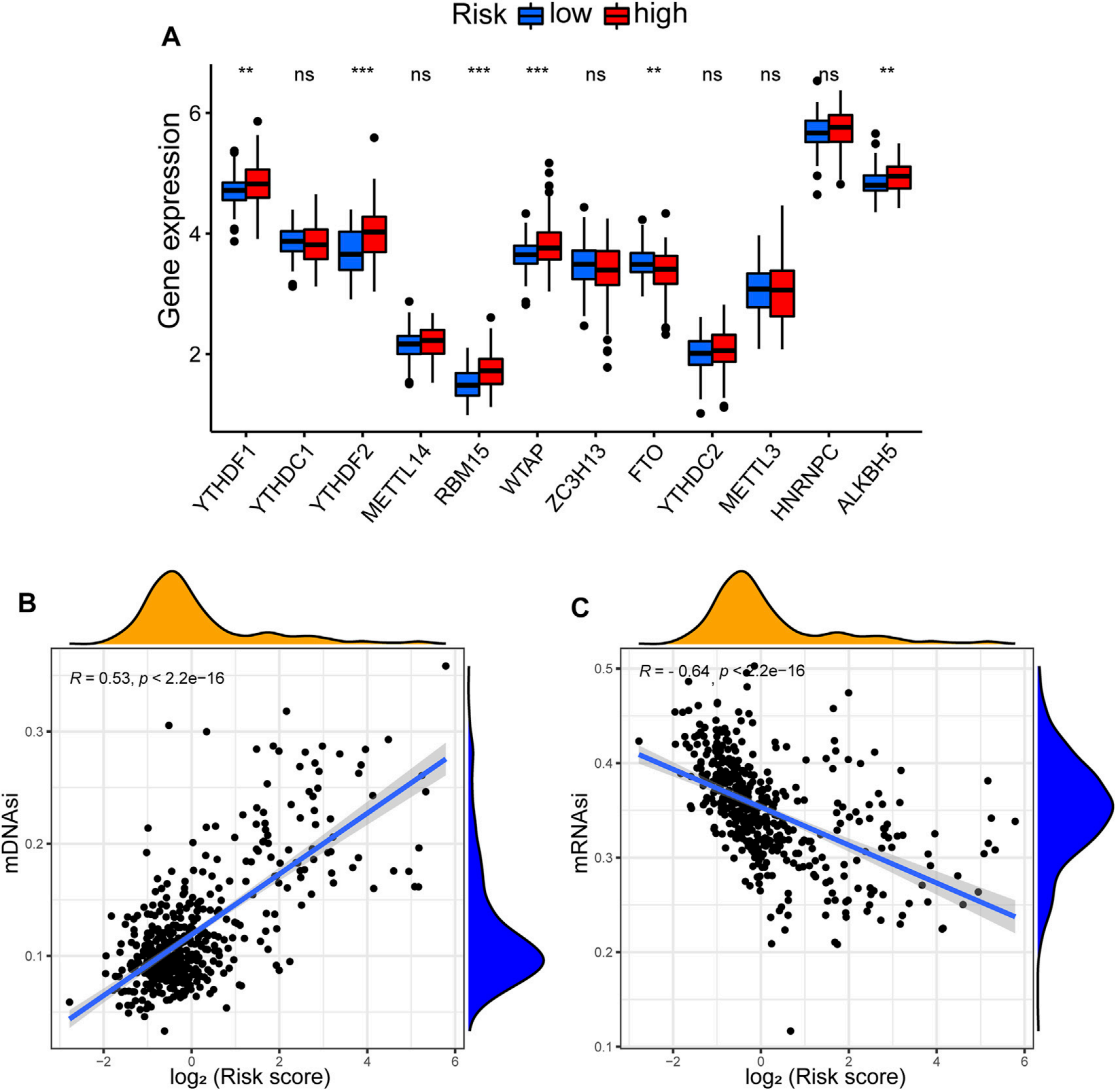
Correlation between GInLncSig and *N* ^6^-methyladenosine (m^6^A) and stemness index. **(A)** The expression level of m^6^A-related genes between high- and low-risk groups. **(B)** The relationship between risk score and stemness index based on DNA methylation pattern (mDNAsi). **(C)** The relationship between risk score and stemness index based on mRNA expression (mRNAsi).

We investigated the relationship between the expression of m^6^A-related genes and the risk of our GInLncSig. The expression of m^6^A-related mRNA where significant differences had been found between the high- and low-risk groups in TCGA dataset include YTHDF1, YTHDF2, RBM15, WTAP, FTO, and ALKBH5 (Figure 12A). Cancer stemness could be detected by DNA methylation pattern (mDNAsi) or RNA stemness score based on mRNA expression (mRNAsi) (Malta et al., 2018). Correlation analysis between the risk score of GInLncSig and stemness in TCGA dataset suggested that the risk score was significantly positively correlated with mDNAsi (*p* < 0.001; Figure 12B), but significantly negatively correlated with mRNAsi (*p* < 0.001; Figure 12C).

### Cancer Cell Chemosensitivity

**FIGURE 13:**
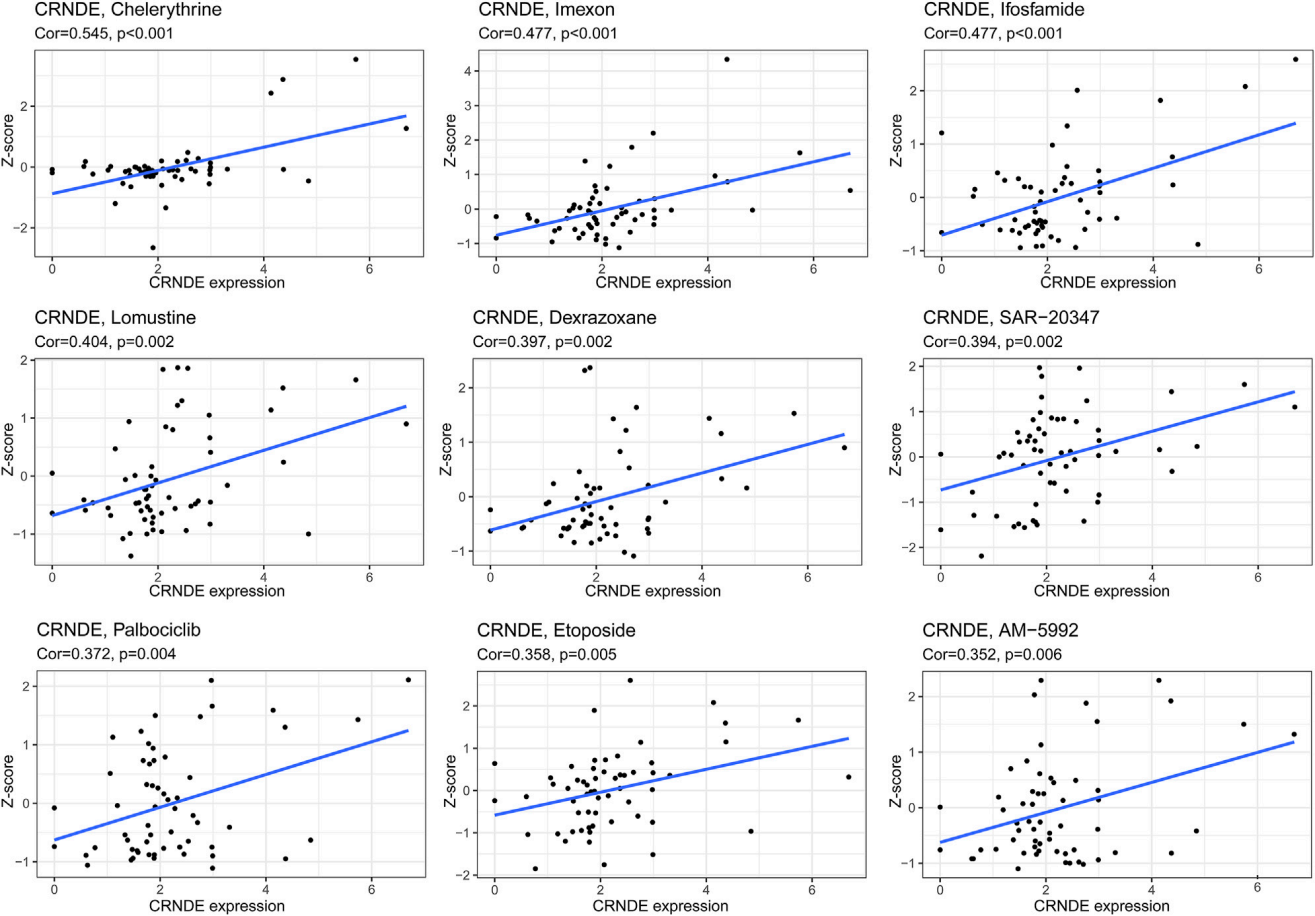
Correlation between CRNDE from GInLncSig and drug sensitivity. The vertical axis shows the Z-scores of drugs. The horizontal axis represents gene expression. The larger the Z-score means, the more sensitive the cancer cell to the drug.

We delved into the expression level of prognostic GInLncRNAs in NCI-60 cancer cell lines and construed the conjunction between their expression levels and sensitivity of chemotherapeutic agents. However, we found that only two GInLncRNAs (CRNDE and TGFB2-AS1) were present in the NCI-60 expression profile, and TGFB2-AS1 expression had many abnormal and missing values. Therefore, we chose only CRNDE to perform the correlation analysis of chemotherapeutic agents. The results suggested that CRNDE correlated to the sensitivity of certain chemotherapeutic drugs (*p* < 0.01) (Figure 13). Overexpression of CRNDE promoted the drug sensitivity of tumor cells to chelerythrine, imexon, ifosfamide, lomustine, dexrazoxane, SAR-20347, palbociclib, etoposide, etc.

## Discussion

Genomic instability mainly results from DNA repair defects. Studies have shown that genomic instability is a driver of neoplasm initiation and that the degree of genomic instability is associated with neoplasm aggressiveness (Capelli et al., 2009; Xie et al., 2011). On the other hand, genomic instability could result in intra-tumor heterogeneity, which is also a significant cause of treatment resistance (Gerlinger and Swanton, 2010). Thus, genomic instability is a hallmark of cancer, and accurately measuring a person’s ability to maintain genomic stability has the potential to evaluate the risk of tumor development (Jalal et al., 2011), whereas quantifying the extent of genomic instability remains a significant challenge.

Currently, some researchers have identified mRNAs and miRNAs associated with genomic instability and developed mRNA or miRNA signatures to predict genomic instability (Mettu et al., 2010; Wang et al., 2017; Biermann et al., 2020). In recent years, lncRNAs have come into medical view as novel biomarkers for tumor diagnosis and prognosis and have also been demonstrated to be associated with genomic stability (Lee et al., 2016; Nguyen et al., 2020; Zhang et al., 2020b). Despite several endeavors by researchers, the exploration of lncRNAs related to genomic instability and their clinical implications in tumors is still in the initial stage. In addition, the relationship between lncRNAs associated with genomic instability and gliomas with a highly heterogeneous prognosis remains understudied. Therefore, we identified a set of GInLncRNAs in LGG and integrated multi-omics data for a comprehensive analysis.

In this study, after comparing the expression profiles of lncRNAs in patients with the top 25% mutation counts and those with the bottom 25% mutation counts, we first found 39 differentially expressed lncRNAs. The entire TCGA samples were then classified into the GS and GU clusters by hierarchical clustering based on 39 differentially expressed lncRNAs. Finally, we performed a differential expression analysis between all the samples of these two clusters and confirmed 52 GInLncRNAs. Enrichment analysis of co-expression network presented that the top 10 mRNA co-expressed with GInLncRNAs were mainly enriched in synaptic transmission activity and regulation in GO analysis and enriched in synapse-related pathways, immune-related pathways, and cancer pathways in KEGG analysis. The regular transmission of synaptic signals is inseparable from genomic stability. Recent studies have shown that genomic instability contributes to inducing and activating immune responses (Reisländer et al., 2019; Zhang et al., 2020a). The GInLncSig model was constructed in the training dataset and consisted of five lncRNAs in our research. CRNDE, AL390755.1, AC025171.5, and TGFB2-AS1 were upregulated in the high-risk group and related to poor prognosis except for AL049749.1. LGG patients with a lower GInLncSig risk score were found to have favorable patient survival, which was further verified in an independent internal validation dataset. We also performed stratified clinicopathological analysis and survival analysis of GInLncRNA CRNDE in an external CGGA dataset. In addition, the GInLncSig model is strongly associated with tumor mutant phenotype and CDC20 expression in LGGs, both of which are essential clues of genomic instability. Noticeably, the GInLncSig was also strongly associated with survival outcomes in various clinical subgroups. Univariate and multivariate analyses revealed that, in addition to age and grade, our GInLncSig model was an independent prognostic factor for OS in patients with LGG.

According to the risk model, the IDH1 mutation ratio of LGG patients in the low-risk group was significantly higher than that in the high-risk group, indicating that our GInLncSig could catch IDH1 mutation status. Moreover, the GInLncSig can dramatically differentiate the various clinical outcomes of LGG patients with IDH1 mutation patterns. The survival analysis integrating IDH1 mutation status and genomic instability revealed that IDH1 mutation patients with GS had a better prognosis than those with GU, and IDH1 wild-type patients with GU had worse survival, suggesting that IDH1 mutation status combined with genomic instability had more excellent prognostic value than IDH1 mutation pattern alone. Actually, the ROC curve indicated that our GInLncSig model had a better predictive value compared with other previously released lncRNA models. Moreover, the GSEA with the GInLncSig model suggested that the high-risk group is dramatically associated with immune-related signaling pathways, tumor-related signaling pathways, and metabolism-related signaling pathways.

Next, we performed a multi-omics integrative analysis to explore the association between risk score and immune characteristics, m^6^A methylation, stemness index, and drug sensitivity. Investigating the interaction between LGG progression and anti-tumor immunity response is vital for the immunotherapies of this disease. Few studies have examined the relationship between immune features and genomic instability in LGGs. Our study found that the GInLncSig model was significantly correlated with multiple immune cells infiltration, 11 immune functions, and six immune checkpoints. Therefore, the GInLncSig model can predict the expression level of immune checkpoints and is informative for immunotherapy decisions, such as anti-PD-L1 antibody, verified to have clinical activity in different tumors (Chinai et al., 2015). Epigenetic changes are potentially closely associated with genomic instability and susceptible to oncogenic transformation (Schreiner et al., 2013). The m^6^A methylation is one of the most common epigenetic changes and has complicated functions in tumors. However, the relationship between m^6^A methylation and genomic instability in gliomas has rarely been studied. Overexpression of ALKBH5, an m^6^A demethylase, is related to poor prognosis in gliomas (Zhang et al., 2017), consistent with our result. YTHDF1 has been reported to be upregulated in expression in many tumors, such as colorectal cancer and hepatocellular carcinoma, and may be an essential oncogene (Liu et al., 2020). Our result suggested that YTHDF1 also played a tumor-promotor role in LGG.

Cancer stem cells (CSCs) are a population of cancer cells with self-renewal ability, tumor initiation ability, and drug resistance characteristics (Plaks et al., 2015). Cancer progression involves the gradual loss of differentiated phenotype and the acquisition of progenitor and stem cell-like characteristics. The stemness index (mRNAsi) based on mRNA expression can reflect the stemness expression of transcription, while the stemness index (mDNAsi) based on DNA methylation represents epigenetic stemness characteristics. There is a consistent positive correlation between mRNAsi, mDNAsi, tumor histology, and pathological grade for most cancers. However, our research observed a strong positive correlation between mDNAsi and risk score, while mRNAsi showed a clear opposite trend. The high frequency of IDH1/2 mutations in gliomas and the resulting DNA hypermethylation may explain the contrasting results between mDNAsi and mRNAsi (Malta et al., 2018). By NCI-60 database, we analyzed the correlation between chemotherapy drugs approved by the FDA or on clinical trials and CRNDE. The result suggested that elevated expression of CRNDE increased the sensitivity of tumor cells to chelerythrine, imexon, ifosfamide, lomustine, dexrazoxane, palbociclib, and so forth. Lomustine, which blocks dopamine synthesis and plays an essential role in glioma initiation and progression, is undergoing phase III clinical trials in patients with anaplastic astrocytoma (Levin et al., 2018). In mouse models of brainstem glioma and GBM, palbociclib administration has displayed longer-term survival (Hanaford et al., 2016). Therefore, we speculate that CRNDE could predict drug sensitivity and serve as therapeutic targets to overpower chemotherapy drug resistance or adjuvant chemotherapy drug sensitivity. Finally, we combined the GInLncSig and clinical features, including gender, age, and tumor grade, to establish the nomogram in the training set, which could further improve the effectiveness and accuracy of the prediction model.

There are several limitations to this study. First, we only validated the predictive model against one GInLncRNA (CRNDE) in external validation due to the different detection depths in different databases. Second, four GInLncRNAs (AL390755.1, TGFB2-AS1, AC025171.5, and AL049749.1) were described, for the first time, to be of prognostic relevance in LGG, and further *in vitro* and *in vivo* studies are urgently required for a comprehensive understanding of the possible molecular mechanisms in tumorigenesis and progression of LGG.

## Conclusion

In conclusion, the five-GInLnRNA risk signature was identified and considered a novel potential prognostic biomarker for LGG. The signature was confirmed as an independent risk factor and displayed high valence in correlation with immune characteristics, m^6^A, stemness index, and drug sensitivity, providing strong prognostic predictive power and assessing genomic instability for LGG. In addition, we built a nomogram by combining GInLncSig with clinicopathological features to improve prediction efficacy. Taken together, our study provides guiding value for the hierarchical clinical management and therapeutic target for patients with LGG.

## Data Availability

Publicly available datasets were analyzed in this study. These data can be found here: TCGA website (https://portal.gdc.cancer.gov/), the CGGA website (http://www.cgga.org.cn/, DataSet ID: mRNAseq_693), the TIMER website (https://cistrome.shinyapps.io/timer/), and the CellMiner website (https://discover.nci.nih.gov/cellminer/home.do).

## Supplementary Material

Supplementary Figure S1 Oncoplot depicted the distribution of the top 30 mutated genes in LGG samples. The upper plot displayed the cumulative frequencies of mutation for each LGG patient. The lower plot showed the mutated genes and their corresponding types of mutation in each LGG sample. Mutated genes were ranked by their mutation frequency. The right panel displayed the number of variants in each gene.

Supplementary Figure S2 Volcano plot presented 39 differentially expressed lncRNA between samples with the top 25% mutations and samples with the last 25% mutations according to the |Log2(Fold change) > 1.585 and false discovery rate (FDR) adjusted *p* < 0.05.

Supplementary Figure S3 Kaplan–Meier survival curves for GS and GU clusters in TCGA dataset. The patients in GU cluster displayed significantly worse OS than those in GS cluster (log-rank test, *p* < 0.001).
