# Supplementary figures and images for: Multi-Omics Analysis Based on Genomic Instability for Prognostic Prediction in Lower-Grade Glioma
Source: Front Genet. 2022 Jan 5;12:758596. doi: 10.3389/fgene.2021.758596 (PMC8766732; doi:10.3389/fgene.2021.758596)

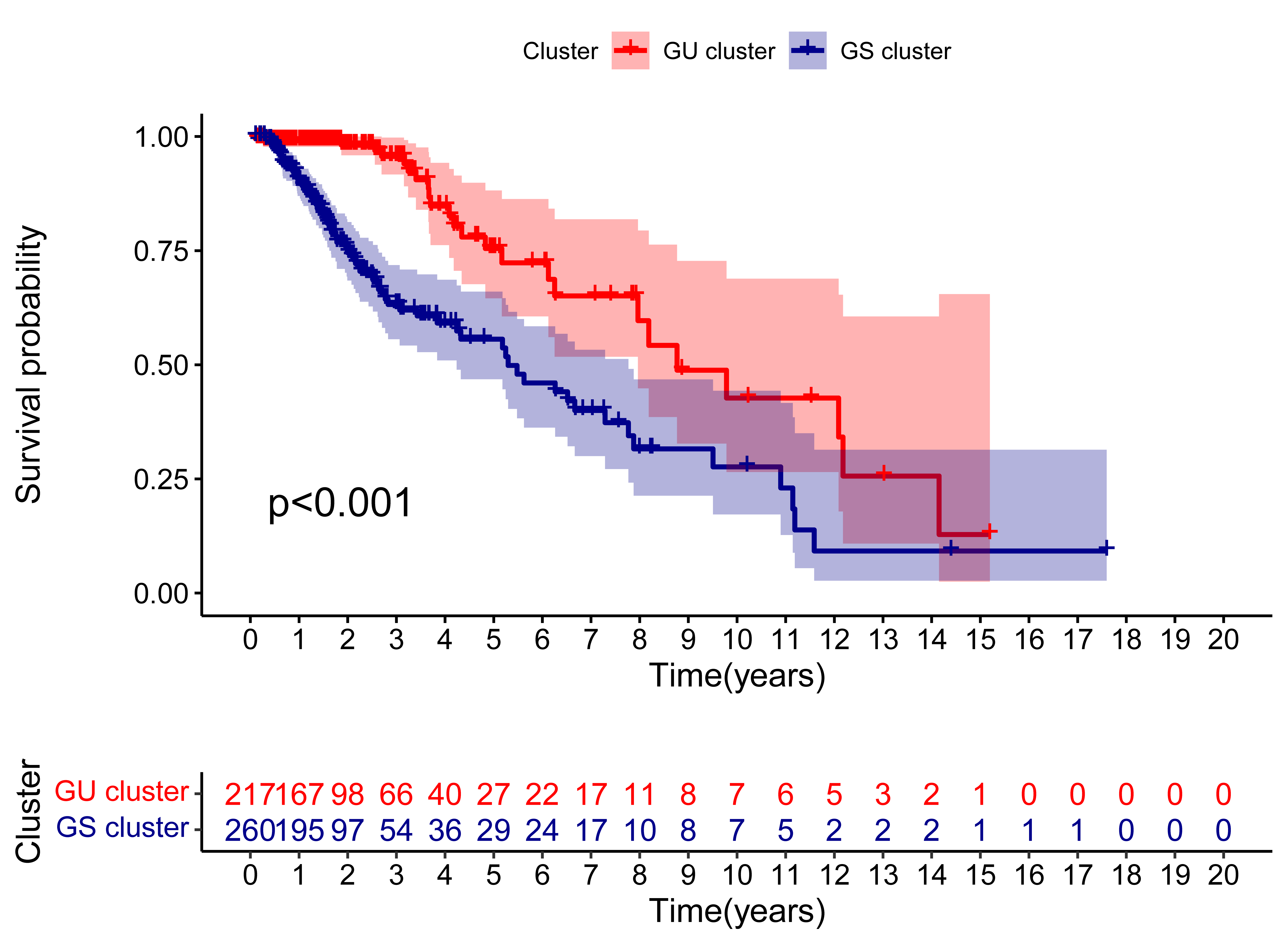

Supplement: Supplementary file 3 [file Image3.TIF]

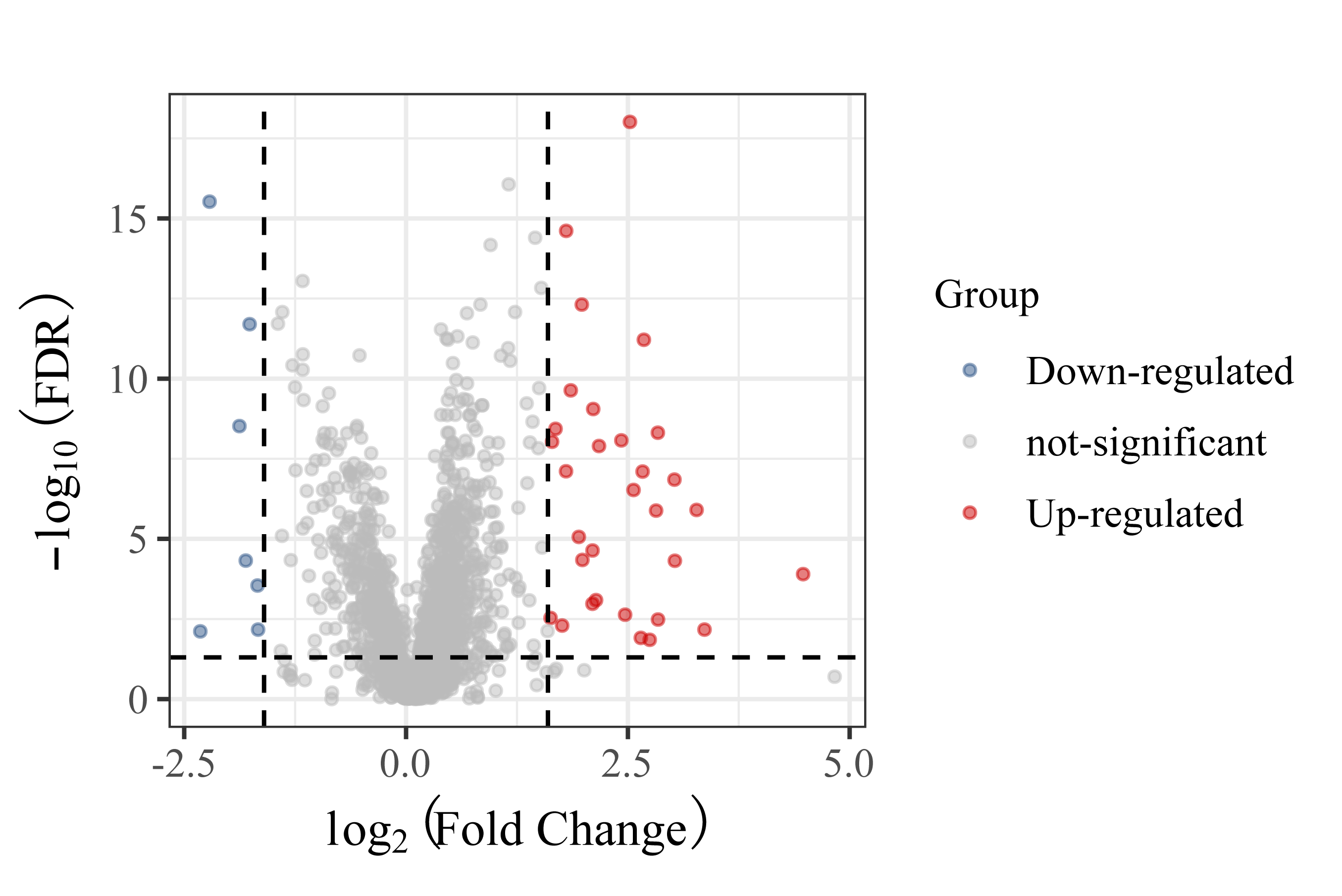

Supplement: Supplementary file 5 [file Image2.TIF]

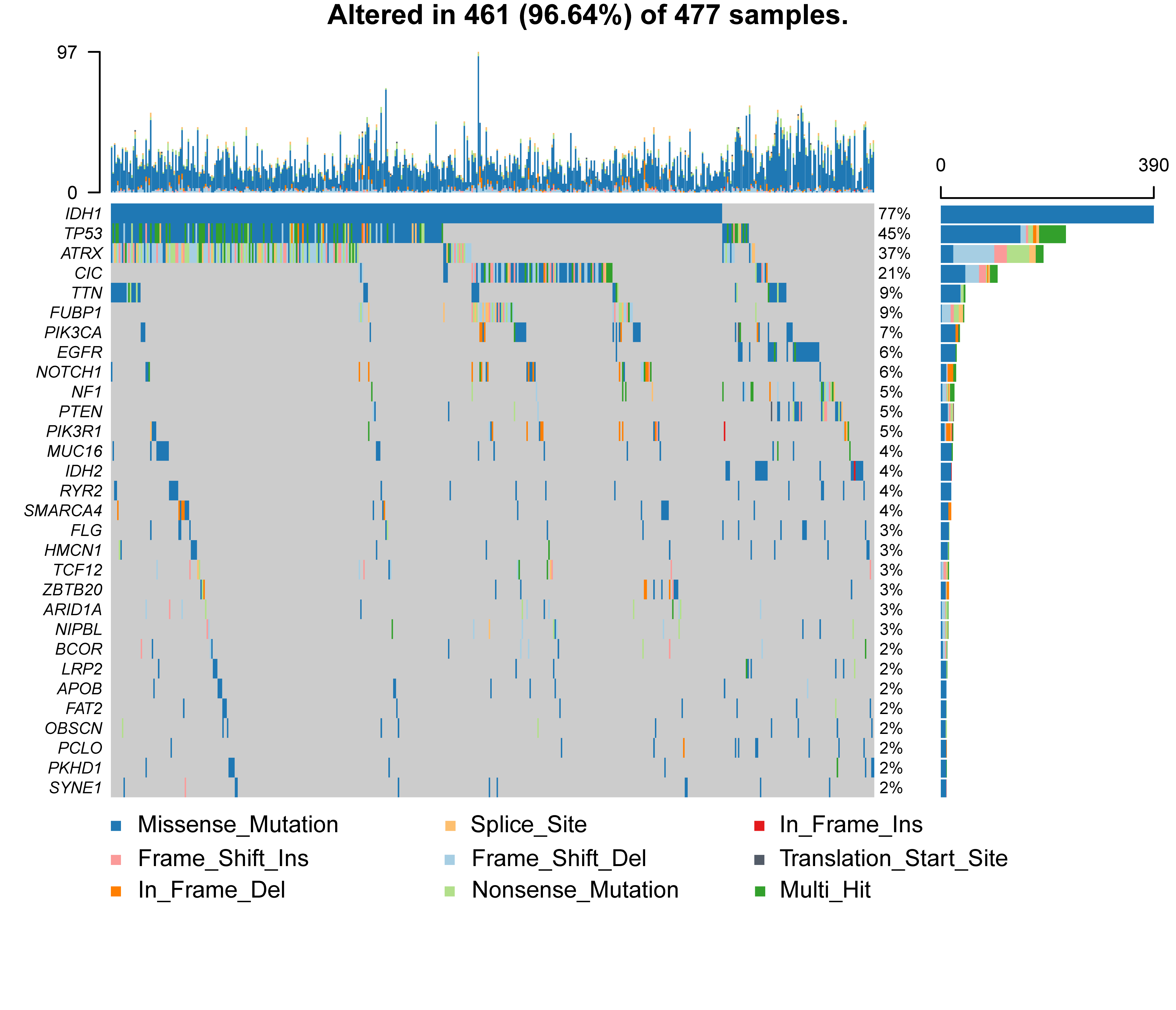

Supplement: Supplementary file 7 [file Image1.TIF]
